# Non optimal temperatures and chronic cardiovascular diseases

**DOI:** 10.1007/s00281-026-01085-w

**Published:** 2026-09-24

**Authors:** Thomas Münzel, Marin Kuntic, Jos Lelieveld, Michael Molitor, Omar Hahad, Paul Stamm, Andreas Daiber, Alexandra Schneider

**Affiliations:** 1 Department of Cardiology, University Medical Center Mainz, Johannes Gutenberg University, Langenbeckstrasse 1, 55131 Mainz, Germany; 2 German Centre for Cardiovascular Research (DZHK), Partner Site Rhine- Main, Mainz, Germany; 3 Atmospheric Chemistry Department, Max Planck Institute for Chemistry, Mainz, Germany; 4 Institute of Epidemiology, Helmholtz Zentrum München-German Research Center for Environmental Health (GmbH), Neuherberg, Germany

## Abstract

Extreme temperatures are emerging as major climate-sensitive drivers of chronic cardiovascular disease (CVD). Global warming has increased the frequency, duration, and intensity of heatwaves, while substantial cold-related risk persists, particularly in poorly insulated and socioeconomically vulnerable settings. Together, non-optimal temperatures account for millions of deaths annually, with a large and growing share attributable to CVD. In this narrative review, we synthesize epidemiological, mechanistic, and systems-level evidence linking heat and cold exposure to chronic cardiovascular outcomes, with a focus on ischemic heart disease, stroke, heart failure, and arrhythmias. We first outline key temperature metrics, shifting climate patterns, and challenges in exposure assessment, including the roles of humidity, diurnal variability, and indoor versus outdoor environments. We then summarize the epidemiology of temperature, CVD associations across diverse regions, highlighting U- and J-shaped exposure-response curves and disproportionate impacts in low- and middle-income countries, older adults, and socioeconomically disadvantaged communities. Mechanistic sections examine how heat and cold drive autonomic imbalance, endothelial dysfunction, oxidative stress, inflammation, haemoconcentration, changes in coagulation, renal injury, and myocardial oxygen supply–demand mismatch. We further discuss modifying effects of air pollution, wildfires, dust storms, and medication use, including clinically relevant drug-heat interactions in patients with chronic CVD. Finally, we review heat-health and cold-health action plans, health-system resilience frameworks, and emerging research gaps spanning exposure science, mechanistic studies, vulnerable populations, and policy evaluation. Recognising non-optimal temperature exposure as a modifiable environmental risk factor for chronic CVD is essential. Integrating climate-informed prevention, clinical care, and adaptation strategies offers substantial and timely opportunities to reduce cardiovascular burden in a warming and increasingly unstable climate.

**Graphical Abstract:**

Graphical abstract illustrating how non-optimal temperatures, including heatwaves, cold spells, temperature variability, and humidity, contribute to chronic cardiovascular disease through vulnerable populations, interacting environmental exposures, and biological mechanisms such as autonomic dysfunction, endothelial dysfunction, oxidative stress, inflammation, thrombosis, renal stress, and circadian disruption. The figure also highlights key chronic cardiovascular outcomes and potential mitigation/adaptation strategies to reduce health risks. **Note**: This graphical abstract was generated with the assistance of ChatGPT.

**Figure:**
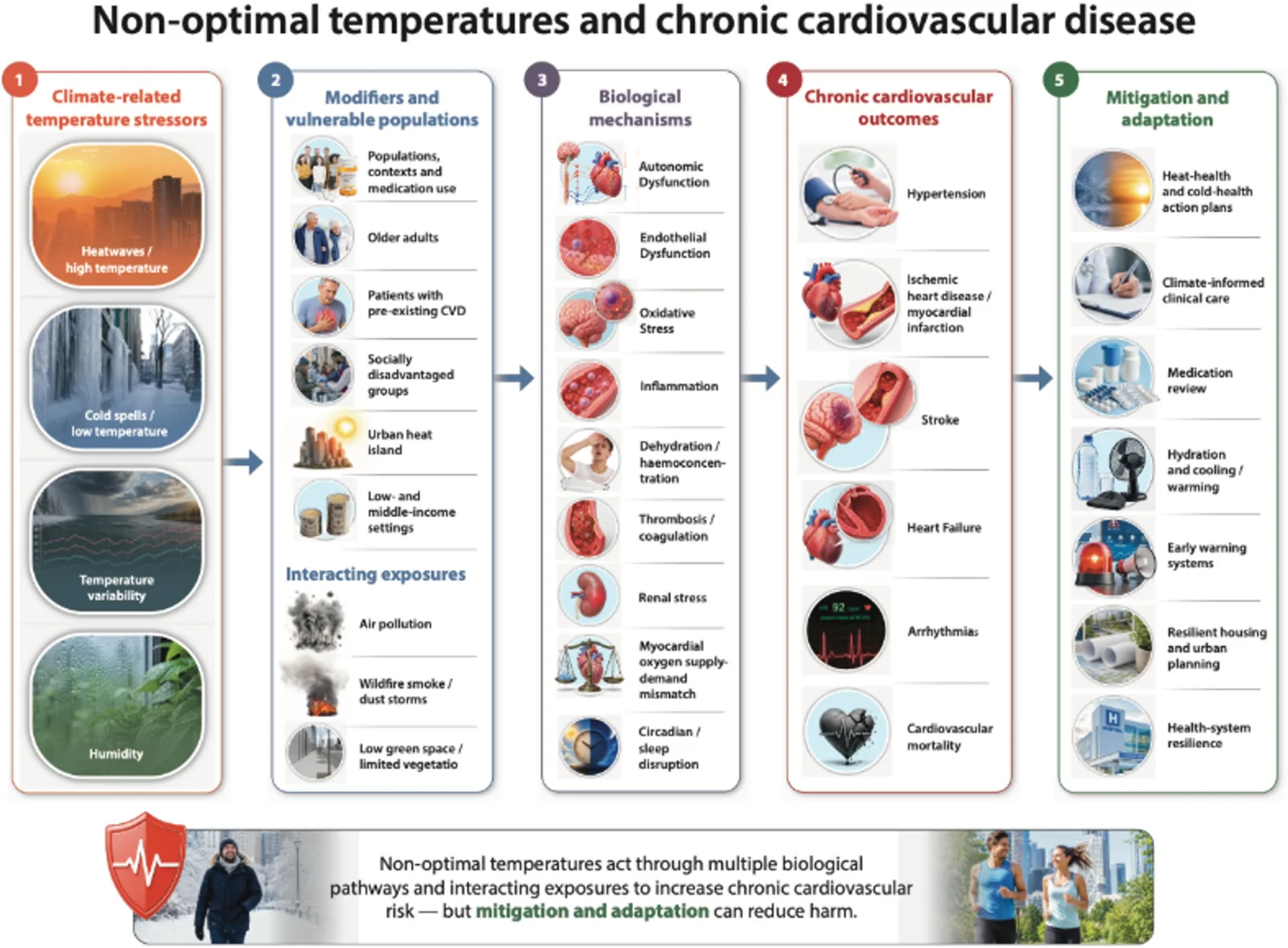

## Introduction

**Fig. 1:**
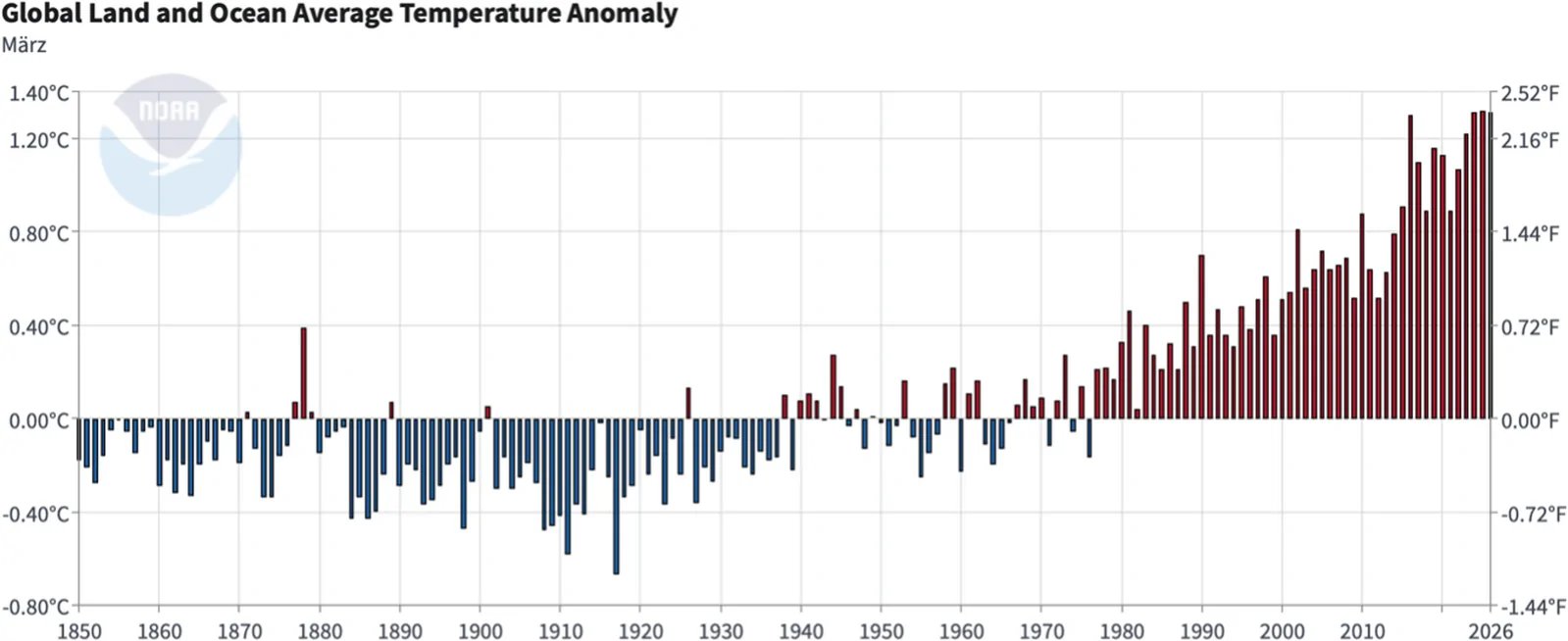
Global Warming Trend and Seasonal Temperature Shift Over Time. This figure illustrates the long-term progression from negative to positive global temperature anomalies, overlaid on a landscape transitioning from winter (left) to summer (right). Blue bars represent annual temperature anomalies below the historical baseline, highlighting decades dominated by cooler-than-average years. Orange and red bars indicate anomalies above the baseline, demonstrating the rapid rise in global temperatures in recent decades. The increasing height and intensity of the orange/red-coloured bars reflect the acceleration of anthropogenic warming, with recent years exceeding + 2 °C above reference levels in some regions [2]

Global climate change has emerged as one of the most significant environmental determinants of human health in the 21 st century. The Earth’s average surface temperature has risen by approximately 1.2 °C since the late 19th century, with most of this warming occurring within the last several decades, making 25 of the hottest years on record occur since 2000 [1] (Fig. 1).

This rapid warming has been accompanied by an unprecedented frequency, intensity, and duration of extreme temperature events, including prolonged heatwaves, severe cold spells, and climate-related natural disasters such as storms, wildfires, droughts and floods. Between 1998 and 2017, climate- and geophysical-related disasters caused an estimated 1.3 million deaths and affected 4.4 billion people worldwide [3]. These trends are projected to accelerate as global temperatures continue to rise and populations, particularly those with multiple cardiometabolic risk factors, become increasingly vulnerable [4].

According to the World Health Organisation, approximately 3.6 billion people live in regions considered highly vulnerable to climate impacts [5]. Such populations, often concentrated in low-income or marginalised communities, face multiple and overlapping hazards, including rises in diarrheal diseases, vector-borne infections such as malaria and dengue, coastal flooding, and childhood stunting. Older adults, who frequently have reduced thermoregulatory capacity and a higher burden of chronic disease, are disproportionately affected. Socioeconomic inequalities, limited access to healthcare, and regional disparities in adaptive capacity compound these climate-related vulnerabilities.

Non-optimal temperatures (NOT) are now considered among the leading risk factors of mortality worldwide [6]. A global analysis showed that 9·4% of all deaths can be attributed to both cold and hot NOTs, corresponding to about 5 million deaths [7]. In most epidemiological studies, excess cold deaths far outnumber heat deaths. In that same global analysis, of the 9·4% attributable temperature-related deaths, 8·5% (range 6·2–10·5%) were cold-related and only 0·9% (range 0·6–1·4%) were heat-related [7], which corresponds to approximately 4·6 million deaths from cold and about 489 000 from heat, a ratio of roughly 9:1 of cold versus heat. This pattern is also consistent in regional studies [8, 9]. Heat-related excess mortality is most pronounced in Europe, whereas the highest cold-related mortality burdens occur in Sub-Saharan Africa. This review nevertheless emphasises heat stress because, unlike the current cold-dominated burden, it is the component of temperature-related risk that is changing fastest: climate warming of 1.5–3 °C is projected to increase heat-related mortality by 0.5–2.5%, while population ageing largely cancels out the modest gains from milder winters [10]; with projections for European cities, including those in Central Europe, indicating that rising heat-related deaths will consistently outweigh the comparatively modest decline in cold-related mortality over the coming decades [11]. Across all regions, individuals with pre-existing cardiovascular conditions suffer disproportionately, reflected by increases in emergency department utilisation, ambulance calls, and hospital admissions during temperature extremes [4].

Compounding the direct effects of temperature, adverse environmental exposures, particularly air pollution, interact synergistically with heat stress, exacerbating cardiovascular risk and contributing to more severe outcomes [12]. Demographic trends, including population growth, rapid urbanisation, ageing societies, and widening socioeconomic disparities, further increase the proportion of people exposed to harmful temperature conditions [13]. Together, these forces shape a global environment in which climate-related cardiovascular vulnerability is rising sharply.

The cardiovascular system is uniquely sensitive to temperature stress because thermoregulation relies heavily on dynamic cardiovascular adjustments [14]. Cold exposure increases sympathetic activity, systemic vascular resistance, blood pressure, and blood viscosity, all of which impose additional strain on individuals with coronary artery disease, hypertension, heart failure, or cerebrovascular disease. Heat, conversely, leads to peripheral vasodilation, dehydration, tachycardia, electrolyte imbalances, and reduced renal perfusion, processes that may precipitate ischemia, arrhythmias, and heart failure decompensation. Epidemiological studies in multiple regions consistently demonstrate U- or J-shaped temperature, mortality curves, with significant excess cardiovascular risk at both low and high extremes [4, 7]. Even minor deviations from a region’s minimum mortality temperature are associated with increased cardiovascular events.

This narrative review is designed to synthesise current scientific understanding of the cardiovascular consequences of exposure to non-optimal temperatures with a focus on patients with chronic coronary artery disease. It describes the epidemiological burden, identifies population-level vulnerability factors, and elucidates mechanistic pathways, including the roles of autonomic imbalance, endothelial dysfunction, inflammation, oxidative stress, and hemodynamic changes, that link thermal stress with cardiovascular pathology. Furthermore, the review examines interactions between temperature and cardiovascular medications, outlines specific challenges in acute and intensive cardiac care during extreme temperatures, and provides practical guidance for clinicians on implementing heat- and cold-health action plans. Finally, it discusses climate adaptation and mitigation strategies, particularly those relevant to healthcare systems, to better protect patients amid rapid environmental change.

## Temperature metrics, climate trends, and exposure assessment

A clear understanding of how temperature exposures affect cardiovascular health rests on three prerequisites: rigorous definitions of thermal metrics, awareness of evolving climate patterns, and reliable exposure assessment. Cardiovascular impact is not determined by absolute temperature alone but by a constellation of concurrent factors, humidity, exposure duration, diurnal variation, seasonality, individual acclimatisation, and co-exposures such as air pollution. Climate change has reshaped these patterns over recent decades and is steadily increasing the global burden attributable to non-optimal temperature exposure.

Non-optimal temperature (NOT) covers a wide spectrum of exposures, from isolated hot days and multi-day heatwaves to sudden cold spells, prolonged moderately cold periods, and pronounced day-to-night swings. While extreme heat and cold are typically defined by location-specific percentile thresholds [7], cardiovascular risk does not begin only at these extremes: epidemiological evidence consistently shows that even modest deviations from a region’s minimum mortality temperature (MMT) translate into measurable increases in cardiovascular morbidity and mortality [15]. Beyond absolute exposure, temperature variability, rapid fluctuations within hours or across days, wide diurnal ranges, and unstable seasonal transitions, has emerged as an independent cardiovascular stressor, disturbing autonomic balance, promoting endothelial stress, and amplifying susceptibility to ischemic events, arrhythmias, and heart failure decompensation [4].

Humidity should not be regarded simply as an amplifier of heat through perceived discomfort but as a bidirectional, context-dependent modifier of thermal cardiovascular stress. High humidity impairs evaporative heat loss, increasing core temperature, cardiac output demand, dehydration risk, and myocardial oxygen demand. Conversely, very low humidity may worsen cardiovascular risk by accelerating fluid loss, promoting mucosal dryness and respiratory irritation, and facilitating co-exposure to dust, wildfire smoke, and particulate matter; in winter, low humidity also enhances viral transmission, indirectly increasing cardiovascular risk through infection-related pathways [12]. Both humid and dry heat may therefore increase cardiovascular vulnerability, albeit through different mechanistic routes, impaired heat dissipation in humid heat versus enhanced dehydration plus inhalational co-stressors in dry heat. These humidity-temperature interactions remain insufficiently characterised and warrant explicit attention in future epidemiological and mechanistic studies.

The metrics used to capture temperature exposure span simple ambient temperature, humidity-adjusted indices (apparent temperature, humidex, heat index), and physiologically grounded composite indices such as the Wet-Bulb Globe Temperature (WBGT), the Universal Thermal Climate Index (UTCI), and the Physiological Equivalent Temperature (PET) [13]. For cold exposure, wind chill indices capture convective heat loss, although cardiovascular-specific physiological models remain less developed. These are complemented by event-based metrics such as heatwaves, cold spells, diurnal temperature range, and cumulative or compound exposures. The definitions, components, strengths, limitations, and cardiovascular relevance of these metrics are summarised in Table 1.

**Table 1:** Temperature Metrics Relevant to Cardiovascular Risk Assessment

Metric	Definition/Calculation	Components Considered	Strengths	Limitations	Cardiovascular Relevance
Ambient temperature (T_air)	Absolute air temperature (°C/°F) measured at standard height (≈ 2 m)	Air temperature only	Widely available; simple; comparable across regions	Ignores humidity, radiation, wind, indoor/outdoor differences	Most commonly used exposure variable in cardiovascular epidemiology
Hot night excess Index	Excess sum of high temperature during the nighttime hours	Hourly air temperature	Quantifies the intensity of nocturnal thermal stress	Requires hourly temperature data	Sustains sympathetic activation, sleep disruption, dehydration
Apparent temperature/Heat Index/Humidex	Empirical combination of air temperature and relative humidity	Temperature + humidity	Easy to compute; used in public-health alerts	Limited validity in elderly and patients with impaired thermoregulation; ignores radiation/wind	Approximates perceived heat stress; useful for warning systems
Wet-Bulb Globe Temperature (WBGT)	Composite index: 0.7 × T_wet-bulb + 0.2 × T_globe + 0.1 × T_air	Temperature, humidity, radiant heat, wind	Integrates multiple thermal stressors; standard in occupational settings	Requires specialized instruments; less validated indoors	Reflects cardiovascular load during heat exposure in outdoor/occupational contexts
Universal Thermal Climate Index (UTCI)	Equivalent temperature derived from a multi-node human energy-balance model	Temperature, humidity, wind, radiation, metabolic and clothing factors	Physiologically grounded; applicable across climates	Computationally demanding; requires high-resolution meteorological inputs	Provides best approximation of human thermal strain for cardiovascular research
Physiological Equivalent Temperature (PET)	Air temperature of a reference indoor environment producing equivalent core/skin temperatures	Body energy balance (core and skin temperatures)	Models individual physiological strain	Assumes standardized clothing/activity; less suited to populations with comorbidity	Estimates cardiovascular and thermoregulatory load
Wind Chill Index	Equivalent temperature combining ambient cold with wind speed	Temperature + wind speed	Captures convective heat loss	Does not include humidity or radiative loss; cardiovascular models underdeveloped	Relevant for cold-related sympathetic activation, vasoconstriction, BP elevation
Minimum Mortality Temperature (MMT)	Location-specific temperature associated with the lowest cardiovascular mortality	Local population mortality data	Population-tailored optimum; basis for “non-optimal temperature” definition	Varies by region, time period, age, comorbidity; requires long mortality time series	Reference point for quantifying heat- and cold-related cardiovascular risk
Extreme heat thresholds	Location-specific high percentiles (≥ 95th or ≥ 99th) of daily temperature distribution	Temperature distribution	Adapts to local acclimatisation; standard in DLNM analyses	Threshold choice influences risk estimates	Defines extreme heat exposure linked to ischemic events, arrhythmia, HF decompensation
Extreme cold thresholds	Location-specific low percentiles (≤ 5th or ≤1st) of daily temperature distribution	Temperature distribution	Population-relative; reproducible	Sensitive to reference period and climate change drift	Defines extreme cold exposure linked to MI, stroke, prothrombotic state
Diurnal Temperature Range (DTR)	Difference between daily maximum and minimum temperature	Intra-day temperature variability	Captures short-term thermal stress	Does not reflect day-to-day variability	Associated with autonomic imbalance, endothelial stress, arrhythmia, HF
Temperature variability (TV)	Standard deviation of temperature within or across days	Hour-to-hour or day-to-day fluctuations	Independent cardiovascular stressor beyond mean exposure	Multiple computational definitions; inconsistent reporting	Linked to ischemic events, arrhythmia, HF exacerbations
Heatwave	≥ 2–3 consecutive days exceeding a defined high-temperature threshold (e.g., ≥ 95th–99th percentile)	Intensity + duration	Captures sustained thermal stress	Definitions vary across studies and regions	Associated with cumulative cardiovascular load and mortality
Excess Cumulative Heat (ECH)	Integrated thermal load above a threshold over the duration of a heat event	Intensity × duration (cumulative)	Reflects total heat burden, not single hot days	Less standardized; requires high-resolution data	Nonlinear association with cause-specific cardiac mortality (Yu et al., 2025)
Compound day–night heatwave	High daytime temperatures combined with persistently warm nights	Day + night persistence	Captures absence of nocturnal recovery	Requires sub-daily data; not yet routinely reported	Sustains sympathetic activation, sleep disruption, dehydration; greater risk than isolated day/night events
Cold spell	≥ 2–3 consecutive days below a defined low-temperature threshold (e.g., ≤5th–1st percentile)	Intensity + duration of cold	Captures sustained cold strain	Definitions heterogeneous	Linked to MI, stroke, BP elevation, prothrombotic state, respiratory infection burden
Personal/wearable thermal exposure	Individual-level measurements from wearable or smartphone-linked sensors	Real-time temperature, humidity, sometimes activity	Captures indoor exposure, mobility, occupational settings, microenvironments	Limited deployment in large cohorts; data harmonisation challenges	Enables exposome-informed cardiovascular epidemiology and precision risk assessment

***Abbreviations***: BP = blood pressure; CVD = cardiovascular disease; DLNM = distributed lag non-linear model; DTR = diurnal temperature range; ECH = excess cumulative heat; HF = heart failure; MI = myocardial infarction; MMT = minimum mortality temperature; PET = physiological equivalent temperature; TV = temperature variability; UTCI = universal thermal climate index; WBGT = wet-bulb globe temperature

Heatwaves and cold spells deserve particular attention because they capture not only the magnitude of thermal stress but also its duration, persistence, and cumulative burden. Heatwaves have become longer, more frequent, and more intense across Europe, North America, Asia, and Australia. Global warming, however, does not eliminate cold-related risk: milder winters may reduce some direct cold exposure, but winter mortality remains driven by respiratory infections, poor housing, fuel poverty, and inadequate heating, particularly in older adults and patients with chronic disease [16].

Traditional definitions based solely on daily maximum or minimum thresholds may underestimate cardiovascular risk by overlooking cumulative exposure, delayed recovery, variability, and day–night persistence. Recent work, therefore, conceptualises heatwaves as multidimensional events that integrate intensity, duration, timing, and cumulative excess temperature. In a study of 2.39 million patients, Yu et al. demonstrated a non-linear association between excess cumulative temperature during heatwaves and cause-specific cardiac mortality, supporting the view that risk rises with the accumulated thermal load of prolonged events rather than with isolated “hot days” [17]. Compound day–night heatwaves, in which persistently warm nights deny the cardiovascular system its nocturnal recovery, are of particular concern, sustaining sympathetic activation, tachycardia, dehydration, sleep disruption, endothelial stress, and myocardial supply–demand mismatch; Wang et al. showed that such compound hot extremes, increasingly frequent due to anthropogenic emissions and urbanisation, pose greater health risks than isolated daytime or nighttime events, particularly among older and female urban residents [18]. Cold spells should likewise be regarded as dynamic events rather than isolated cold days, thereby triggering sympathetic activation, peripheral vasoconstriction, elevated blood pressure, increased afterload, haemoconcentration, and a prothrombotic state.

The temperature-related mortality burden shows pronounced geographical heterogeneity. Mortality is therefore shaped not only by ambient temperature but also by population vulnerability, housing quality, healthcare access, acclimatisation, socioeconomic conditions, and adaptive capacity. Climate-driven extreme events add further complexity: wildfires, droughts, and dust storms, all intensified by rising temperatures, generate hazardous air-quality conditions that synergistically amplify cardiovascular risk [12].

Accurate quantification of individual temperature exposure remains challenging. Most epidemiological studies rely on ambient measurements from fixed weather stations or gridded datasets, which may misclassify personal exposure by failing to capture indoor versus outdoor environments, access to air conditioning or heating, occupational exposures, mobility patterns, and urban heat island effects (where city temperatures exceed surrounding rural areas by 5–10 °C and disproportionately affect low-income and minority populations). Indoor cold exposure may likewise be underestimated, particularly in households facing energy poverty or poor insulation. Wearable sensors and smartphone-linked personal environmental monitors offer opportunities for improved characterisation [19], but their uptake in large cardiovascular cohorts remains limited. Integrating high-resolution exposure models with electronic health records, longitudinal biobanks, and personal sensor data represents a promising direction for exposome-informed cardiovascular epidemiology [20].

Future temperatureCVD studies and early-warning systems should move beyond simple threshold-based definitions and incorporate compound exposures, day–night persistence, cumulative heat or cold burden, short-term variability, indoor exposure, humidity, air-pollution co-exposures, and social vulnerability, a broader framework essential for anticipating future cardiovascular burdens and tailoring adaptation strategies to regional risk profiles.

## Non-optimal temperatures and climate hazards as drivers of cardiovascular risk

The Lancet Countdown on Health and Climate Change has comprehensively documented the cardiovascular impact of extreme temperatures and other climate-related hazards [21]. Heat-related mortality among individuals over 65 years has increased by a record 167% compared with the 1990 s — 102% points beyond what would have been expected without temperature rise [21]. A substantial share of this excess mortality is cardiovascular, reflecting the heightened sensitivity of the cardiovascular system to both heat and cold; meta-analyses consistently identify CVD as the leading cause of death during heatwaves across diverse populations and regions [22, 23].

A recent systematic review of 492 observational studies, most from high- and middle-income countries, illustrates the breadth and limitations of the current evidence base: 392 studies addressed non-optimal temperature (NOT) and ozone, while the remainder examined wildfire smoke and extreme weather events such as hurricanes, dust storms, and droughts [24]. Evidence was judged sufficient for extreme temperatures, ozone, tropical storms, hurricanes, cyclones, and dust storms, but limited for wildfire smoke and inadequate for droughts and mudslides — underscoring critical gaps in global climate-health surveillance [24].

Non-optimal temperatures. Both low and high ambient temperatures substantially increase cardiovascular morbidity and mortality [25, 26], with effect sizes varying by exposure magnitude, duration, and population vulnerability [24]. *Climate warming is narrowing the cold-to-heat mortality ratio*: projections across 800 locations in 50 countries indicate that 1.5 °C, 2 °C, and 3 °C of warming will raise heat-related mortality by 0.5%, 1.0%, and 2.5%, respectively, while population ageing largely offsets the gains from milder winters, leaving cold-related deaths essentially unchanged or slightly increased (0.1–0.4%) [10].

Heatwave studies generally account for duration through threshold-based or comparative definitions. Prolonged heatwaves are particularly harmful because they sustain dehydration, cutaneous vasodilation, tachycardia, sleep disruption, renal strain, and myocardial supply–demand mismatch, with insufficient recovery between hot days posing special risk to patients with coronary disease, heart failure, hypertension, or impaired thermoregulation. Liu et al. reported rising cause-specific mortality from compound heatwaves under climate change [6], and Son et al. observed greater risks for longer, more intense, or early-summer heatwaves in Korean cities [27]. Consistent with these data, heatwaves lasting ≥ 5 days in China were associated with an 18% increase in overall mortality (95% CI 6–31%), rising to 24% in adults ≥ 65 years [28, 29].

Regional patterns require careful interpretation. In warm-climate populations, low absolute cold exposure does not equate to low vulnerability: limited heating infrastructure, poor insulation, and reduced cold-risk awareness can produce substantial cardiovascular stress at only moderately low temperatures. Cold-related mortality may therefore remain considerable, and in many regions still exceed heat-related mortality, highlighting the importance of distinguishing meteorological exposure from population vulnerability and adaptive capacity. Hitherto, extreme temperature events have been regarded as transient threats, but with intensifying climate change, younger generations face unprecedented lifetime exposure with progressively shorter return periods, raising the possibility of cumulative health burdens that are currently underestimated and remain to be quantified.

Beyond ambient temperature itself, several co-occurring environmental hazards further modify cardiovascular risk during climate extremes: ground-level ozone, particulate matter from dust storms, and the disruption of essential infrastructure caused by extreme weather events.

### Ozone

Ground-level ozone interacts synergistically with high temperatures, with each exposure amplifying the cardiovascular risks posed by the other [24]. This combined effect is particularly relevant during heatwaves, when stagnant atmospheric conditions promote ozone accumulation.

### Particulate matter and dust storms

Dust storms generate particulate matter across multiple size fractions (PM₁₀, coarse PM, and PM₂.₅). A systematic review and meta-analysis found a 0.27% increase in all-cause mortality on dust-event days [30], and an Asian meta-analysis reported a 2.33% rise in combined respiratory and circulatory mortality [31]. In Taipei, dust-event days were associated with a 26% increase in cardiovascular emergency admissions, including 35% and 20% rises in ischemic heart disease and stroke visits, respectively [32]. Additional studies report elevated risks of ischemic and hemorrhagic stroke and heart-failure admissions linked to dust exposure [33].

### Extreme weather events

Hurricanes, tropical storms, floods, and prolonged power outages produce cardiovascular risks that may persist for months after the initial disaster, primarily by disrupting access to cooling, heating, medications, and healthcare [24]. Older adults, patients with chronic disease, and socially marginalised communities are disproportionately affected.

## Immune system involvement in temperature-related cardiovascular injury

**Fig. 2:**
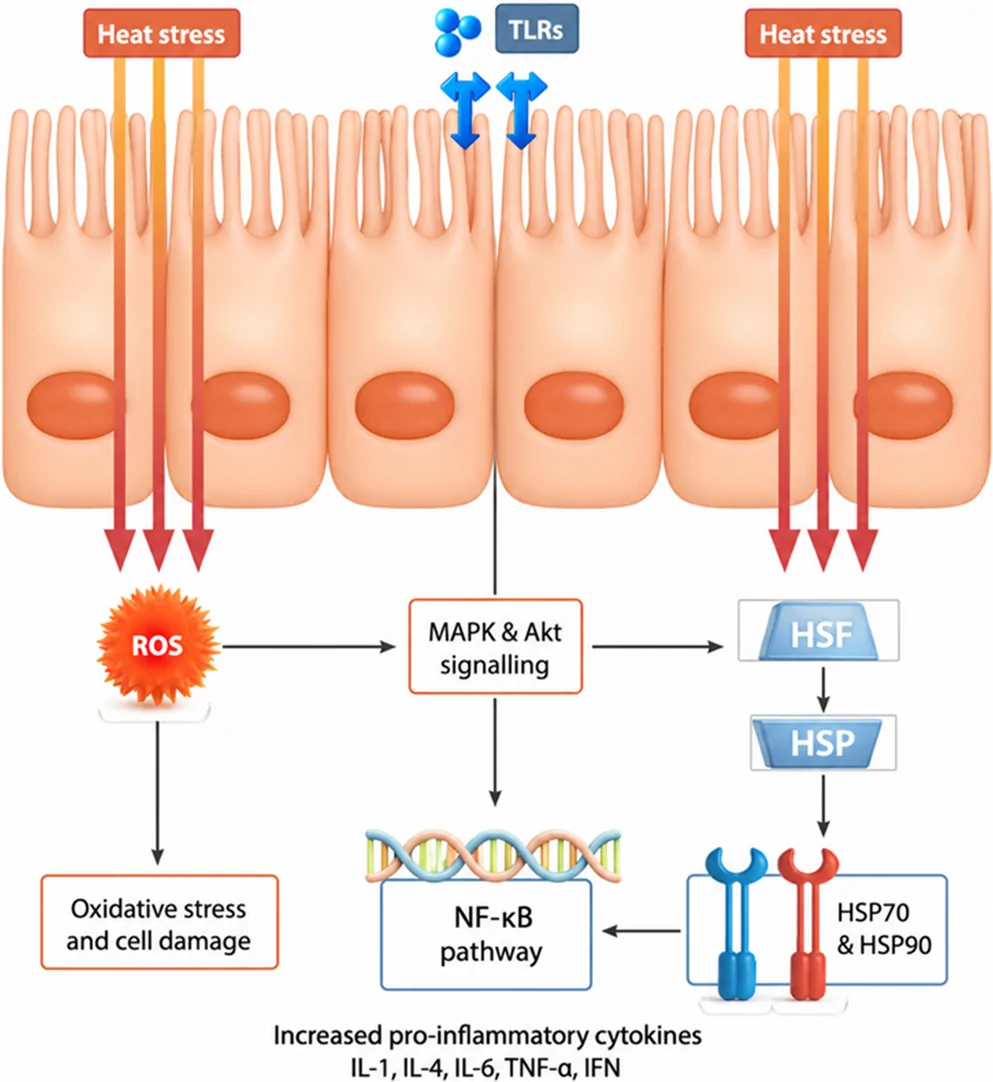
Heat stress increases the production of ROS, activating the heat shock response pathway, resulting in the release of proinflammatory cytokines. **Akt** – Protein kinase B; **HSF** – Heat shock factor; **HSP** – Heat shock protein; **HSP70** – Heat shock protein 70; **HSP90** – Heat shock protein 90; **IFN** – Interferon; **IL-1** – Interleukin-1; **IL-4** – Interleukin-4; **IL-6** – Interleukin-6; **MAPK** – Mitogen-activated protein kinase; **NF-κB** – Nuclear factor kappa-light-chain-enhancer of activated B cells; **ROS** – Reactive oxygen species **TLR(s)** – Toll-like receptor(s); **TNF-α** – Tumor necrosis factor alpha

The immune system is increasingly recognized as a central effector linking environmental stressors to cardiovascular disease [34]. Chronic low-grade inflammation, monocyte and macrophage activation, and pro-inflammatory cytokine signalling drive endothelial dysfunction, atherogenesis, plaque destabilisation, and thrombosis [35]. Monocytes and macrophages are particularly important in this context because they infiltrate the arterial wall, take up oxidised lipids to form foam cells, and secrete matrix-degrading enzymes and pro-inflammatory mediators that drive plaque growth and rupture, while their recruitment and phenotypic switching after myocardial infarction and in heart failure directly shape infarct healing and adverse cardiac remodelling [36] Extreme temperatures perturb this immune homeostasis through distinct but converging pathways. Heat exposure increases the production of ROS that in turn triggers heat-shock protein release, NLRP3 inflammasome activation, and gut-barrier disruption with endotoxin translocation[34], generating a systemic pro-inflammatory and prothrombotic state that closely resembles low-grade sepsis and contributes to cardiovascular collapse during heatstroke [37, 38] (Fig. 2). Notably, heat-shock protein induction is not solely a consequence of ROS signalling: heat directly denatures and aggregates intracellular proteins, and it is this accumulation of misfolded protein aggregates, sensed by molecular chaperones, that constitutes the principal trigger for the heat-shock response [39] Cold exposure, by contrast, elicits sympathetic-driven leukocytosis, redistribution of innate immune cells, and elevated pro-inflammatory cytokines (IL-6, TNF-α), while simultaneously altering adaptive T-cell responses and increasing susceptibility to respiratory infections that further amplify cardiovascular events [40, 41]. These thermally driven immune perturbations interact with shared pathways activated by air pollution and HPA-axis stress, producing a sustained inflammatory milieu that may explain the persistent excess in cardiovascular morbidity and mortality observed during and after extreme temperature events [42].

## How does non-optimal temperature contribute to cardiovascular events?

**Fig. 3:**
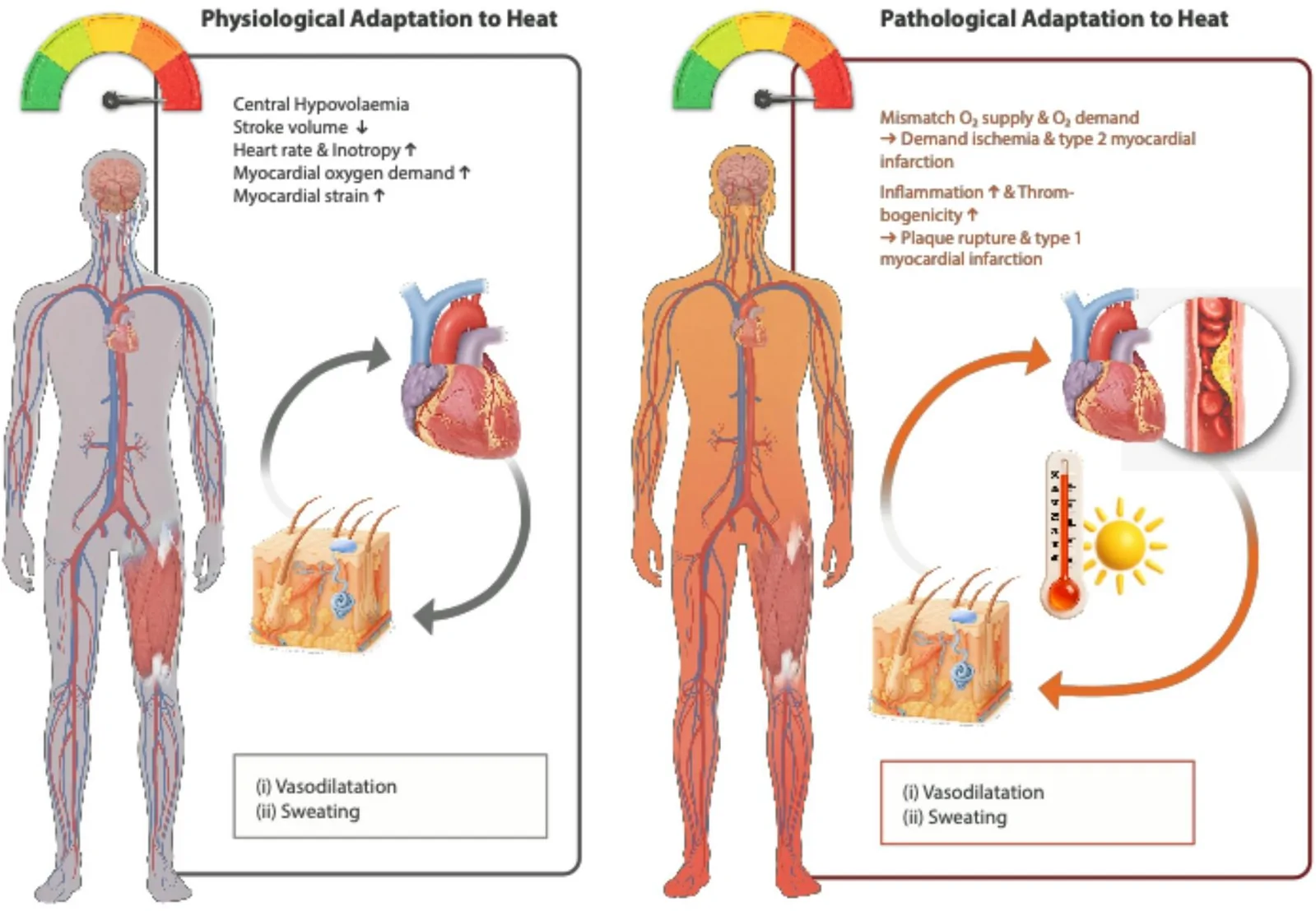

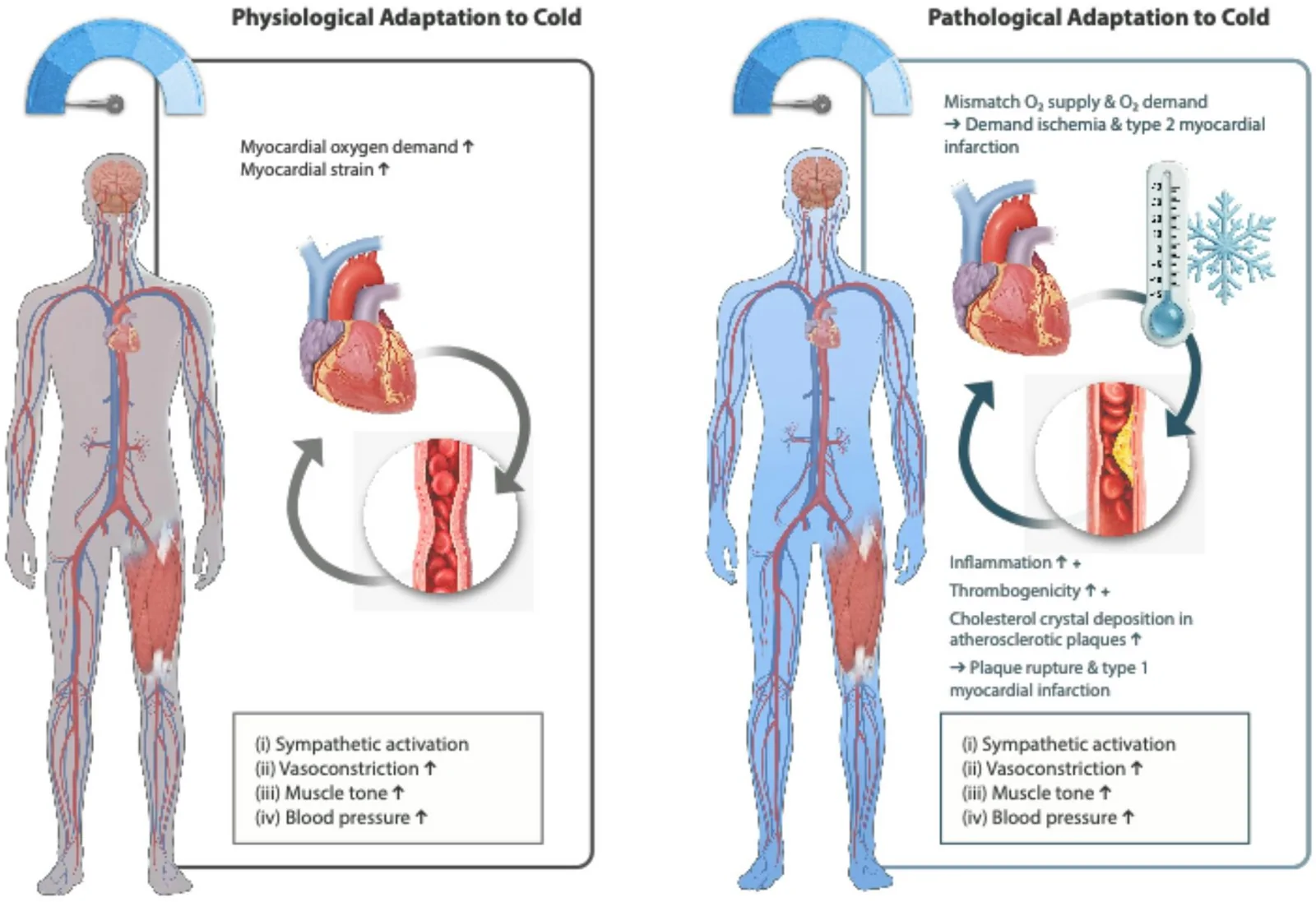
Principal mechanisms involved in non-optimal temperature-mediated cardiovascular events. Upper panel represents physiological and pathological adaptation to heat and lower panel represents physiological and pathological adaptation to cold (adapted with permission from [45])

The human body counters heat stress primarily through two coordinated responses: redistribution of blood flow toward the skin (cutaneous vasodilation) to dissipate heat from the muscular core, and active sweating, which removes body heat through evaporative cooling [43]. These responses are orchestrated centrally by the brain, which integrates afferent signals from temperature-sensitive nerve endings in the skin and deep tissues [44]. Non-thermal modulators, such as dehydration, metaboreceptor activation by metabolic by-products of working muscles, and circulating cytokines, further fine-tune this thermoregulatory control [44]. Although these homeostatic mechanisms normally limit the rise in core temperature, their efficiency varies considerably between individuals and is markedly compromised by pre-existing medical conditions, with potentially deleterious cardiovascular consequences. The principal mechanisms underlying NOT-mediated cardiovascular events are summarised in Fig. 3.

- **Stage 1 — Initial thermoregulatory load (mild to moderate heat exposure).** Cutaneous vasodilation immediately increases cardiac output demand while simultaneously lowering ventricular filling pressure [46], forcing the heart to contract harder and faster and thereby raising myocardial oxygen demand. In patients with underlying cardiac disease, this can precipitate a mismatch between elevated demand and impaired coronary supply; when sustained, the imbalance may culminate in myocardial ischemia, infarction, or cardiovascular collapse [47]. Older adults are particularly susceptible: during extreme heat events, cardiovascular causes account for more heat-related deaths than any other complication [13, 48].
- **Stage 2 — Progressive volume loss and circulatory strain (sustained or intensifying heat).** As heat exposure continues, profuse sweating depletes circulating blood volume and intensifies cardiovascular load [49]. The resulting dehydration can precipitate acute kidney injury and, in cases of repeated exposure, especially among outdoor workers, promote renal fibrosis and chronic kidney disease, both of which further aggravate cardiovascular disease and are frequently observed during or in the aftermath of hot-weather episodes [50].
- **Stage 3 — Thermoregulatory failure and heat stroke (severe heat exposure).** When heat exposure exceeds thermoregulatory capacity, core temperature rises uncontrollably and may culminate in potentially fatal heat stroke [38]. Core temperatures of 39–40 °C, combined with ischemia and oxidative stress from extensive blood-flow redistribution, trigger widespread cellular and tissue injury, with the brain, heart, kidneys, intestines, liver, and lungs being most vulnerable [50].
- **Stage 4 — End-organ injury (endothelial leakage**,** oedema**,** ARDS).** At this stage, endothelial dysfunction, increased microvascular permeability, and systemic inflammation drive multi-organ damage. Exertional heat stroke in particular can cause pulmonary oedema and acute respiratory distress syndrome, especially in patients with pre-existing respiratory conditions [51]. Hyperventilation arises during heat stress as a thermoregulatory response, so-called thermal tachypnoea, through which increased ventilation enhances respiratory heat and water loss; it is further driven by direct stimulation of the respiratory centre by elevated core and blood temperatures, by the rise in metabolic rate associated with hyperthermia, and by compensatory respiratory drive in response to heat-induced metabolic acidosis [52]. Together with this hyperventilation and the worsened air quality typical of heatwaves, these mechanisms account for substantial heatwave-related morbidity and mortality, second only to cardiovascular disease [13].
- **Stage 5 — Long-term consequences.** Heat-induced injury remains hazardous even after the body has been cooled. Although most heat-related hospital admissions occur within 24 h of the event, the consequences, including persistent cognitive impairment and chronic organ dysfunction, may extend for years, sustaining an elevated risk of death for decades after the initial insult [53].
- **Cold exposure.** Cold exposure, by contrast, activates the sympathetic nervous system, producing constriction of cutaneous and skeletal-muscle vasculature. While this response helps conserve, and in some cases generate, body heat, it also raises blood pressure, increases afterload, and elevates myocardial oxygen demand, all of which can exacerbate underlying heart failure. Like heat, cold also induces a hypercoagulable state, characterised by increased blood viscosity, haemoconcentration, and clotting abnormalities [54].

## Non-optimal temperatures and cardiovascular events

### Ischemic heart disease

Ischemic heart disease (IHD) remains the dominant cause of cardiovascular mortality worldwide, and both extreme heat and cold now emerge as significant, climate-sensitive triggers of acute ischemic events. Although the detrimental cardiovascular effects of cold exposure, particularly its association with elevated rates of myocardial infarction (MI), have been recognised for decades, the impact of heat on acute MI has gained increasing attention in recent years as global temperatures rise. The most comprehensive evidence comes from the Multi-Country Multi-City (MCC) collaborative network, which analysed temperature–cardiovascular associations across 567 cities in 27 countries and included more than 32 million cardiovascular deaths. Among these were 11,745,880 ischemic heart disease deaths, 9,351,312 stroke deaths, 3,673,723 heart-failure deaths, and 670,859 arrhythmia deaths, recorded between 1979 and 2019 [4].

**Fig. 4:**
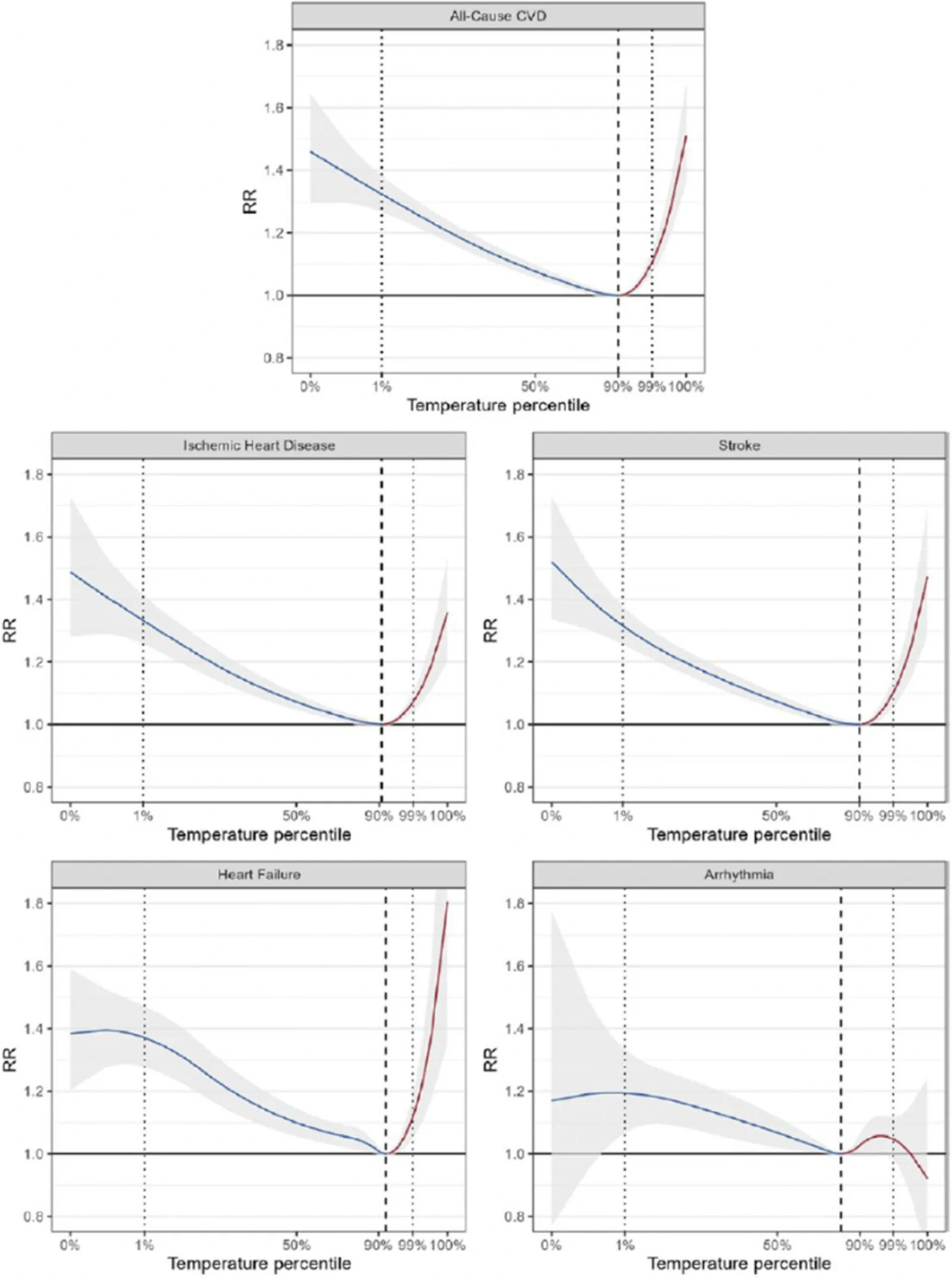
Non-linear exposure–response relationships between temperature percentiles and cardiovascular outcomes. The pooled temperature, mortality associations derived from the Multi-Country Multi-City Collaborative Network (567 cities, 27 countries) illustrate U-shaped or J-shaped curves for all-cause cardiovascular disease (CVD), ischemic heart disease, stroke, heart failure, and arrhythmias. Each panel shows the relative risk (RR) across the full temperature distribution, expressed as percentiles for each location. The minimum mortality temperature (MMT) is plotted at the nadir of the curve, representing the temperature associated with the lowest mortality risk. Extreme cold (1st percentile) and extreme heat (99th percentile) are marked with vertical dashed lines. Cold exposure (< MMT) is associated with a gradually increasing risk across all outcomes, particularly for ischemic heart disease and stroke. Heat exposure (> MMT) produces a steeper rise in risk for ischemic heart disease, stroke, and heart failure, whereas arrhythmia patterns show greater uncertainty due to wider confidence intervals. Shaded areas represent 95% confidence intervals derived from distributed lag non-linear models. These results highlight the substantial health burden of both cold- and heat-related cardiovascular mortality and reinforce the importance of temperature as a major environmental determinant of cardiovascular risk (with permission from [4])

The MCC data revealed a clear non-linear association between temperature and IHD, with increased risk at both hot and cold extremes. Below the minimum mortality temperature (MMT), the risk of ischemic events rose steadily with falling temperature; above the MMT, the slope was steeper for heat, especially for outcomes such as heart failure. For extreme heat (99th percentile versus MMT), the pooled relative risks (RRs) for death from ischemic heart disease, stroke, and heart failure were 1.07 (95% CI: 1.04–1.10), 1.10 (95% CI: 1.06–1.15), and 1.12 (95% CI: 1.05–1.19), respectively. For extreme cold (1st percentile), the RRs were substantially higher at 1.33 (95% CI: 1.26–1.41), 1.32 (95% CI: 1.26–1.38), and 1.37 (95% CI: 1.28–1.47). Arrhythmia-related mortality showed greater uncertainty and smaller effect estimates [4]. (Fig. 4).

Despite its scale, the MCC study has significant limitations, including inadequate representation of highly climate-vulnerable regions, specifically South Asia, the Middle East, and much of Africa. Regions with higher baseline cardiovascular risk, limited healthcare access, and rapid urbanisation may therefore have even larger temperature-related burdens than captured in the dataset.

Additional evidence illustrates a growing contribution of heat to MI risk over time. A longitudinal comparison of MI incidence in periods between 1987 and 2000 and 2001–2014 demonstrated that, while cold exposure triggered MI in the earlier period, the relative risk of heat-related MI increased significantly in the later period [55]. This effect was most pronounced among individuals with diabetes or hyperlipidaemia, highlighting the interaction between thermal stress and underlying cardiometabolic risk. Smaller studies show consistent increases in coronary-artery-disease hospitalisations and emergency visits during heatwaves [56]. Meta-analytic data indicate that every 1 °C rise above reference temperature increases the risk of coronary heart disease by approximately 2.8% [9]. Notably, cold-related cardiovascular impacts tend to persist for 1–2 weeks, whereas heat-related spikes often manifest within 48–72 h [57, 58]. As warming trends accelerate, the relative importance of heat-induced ischemic events is expected to increase substantially.

When looking at temperature variability, a study using data from the SWEDEHEART MI registry (2005–2019) found that an upward temperature shift (reflected by a positive temperature deviation of the actual day compared to the preceding seven days) was associated with increased risks of total MI (encompassing all MI types), STEMI, and NSTEMI hospital admissions with odds ratios (OR, 95% confidence intervals [CIs]) of 1.009 (1.005,– 1.013), 1.014 (1.006–, 1.022), and 1.007 (1.001–, 1.012) per 1 °C increase, respectively. Furthermore, a downward temperature shift (reflected by a negative temperature deviation of the actual day compared to the preceding seven days) was associated with increased risks of hospital admissions for total MI with 2 days delay with an OR (95% CI) of 1.003 (1.001–, 1.005), and for STEMI with an OR (95% CI) of 1.006 (1.002–, 1.010) per 1 °C decrease [59].

The same data in addition showed the relevance of cold effects: A decrease of 1-unit in percentile temperature at a lag of 2 to 6 days was significantly associated with increased risks of total MI, NSTEMI, and STEMI, with ORs of 1.099 (95% CI: 1.057–−1.142), 1.110 (95% CI: 1.060–−1.164), and 1.076 (95% CI: 1.004–−1.153), respectively. Additionally, cold spells at a lag of 2 to 6 days were significantly associated with increased risks for total MI, NSTEMI, and STEMI, with ORs of 1.077 (95% CI: 1.037–−1.120), 1.069 (95% 1.020–−1.119), and 1.095 (95% CI: 1.023–−1.172), respectively [60].

### Stroke

Temperature extremes also influence the incidence and severity of stroke. A meta-analysis of more than 2 million stroke events found that a 1 °C increase in ambient temperature increased stroke risk by 1.1%, while a 1 °C decrease increased risk by 1.2% [61]. These effects were most pronounced among individuals aged 65 years and older. Earlier analyses yielded mixed findings regarding heat and ischemic stroke; a meta-analysis of 21 studies involving 476,511 patients found no consistent association [62]. However, other studies, such as an analysis from Seoul, reported a significant positive correlation between mean daily temperature and ischemic stroke incidence [63].

Strong evidence about stroke severity arises from the MCC consortium: In an analysis of 3.4 million ischemic strokes and 2.5 million haemorrhagic stroke deaths from 522 cities across 25 countries, both extreme heat and cold were associated with increased mortality from ischemic and haemorrhagic stroke [33]. Extreme hot days (top 2.5%) contributed 9.1 excess deaths per 1,000 ischemic strokes and 11.2 excess deaths per 1,000 haemorrhagic strokes, while extreme cold contributed 2.2 and 0.7 excess deaths, respectively. Countries with lower per-capita GDP showed disproportionately higher heat-related haemorrhagic stroke mortality than wealthier nations, reflecting disparities in adaptation capacity and healthcare infrastructure.

Night-time heat is emerging as an independent risk factor. Recent analyses showed that extremely hot nights (97.5th percentile of nighttime temperature) increased stroke risk, with an odds ratio of 1.14 (95% CI: 1.01–1.32), and that this association was stronger in 2013–2020 than in 2006–2012. Older individuals, women, and patients presenting with milder stroke symptoms were particularly susceptible [64]. Additional evidence from China confirms elevated heat-related mortality risks for total stroke (RR 1.54), ischemic stroke (RR 1.63), and haemorrhagic stroke (RR 1.36) [65].

An effect on stroke that has so far been overlooked is unexpected temperature variability, e.g., cold spells in the summer. A study in southern Germany found a markedly elevated risk of both transient ischemic attacks (TIA) and ischemic strokes associated with exposure to cold spell events during the warm season (May–October), using data from 2006 to 2020. The effects on stroke occurrence persisted for up to three days after exposure. The cumulative odds ratios (ORs) for cold spells using the 2.5th percentile as the air temperature threshold were 1.29 (95% CI: 1.09–1.53) and 1.23 (95% CI: 1.05–1.44) for durations of more than one and two days, respectively. The stratified analysis showed that people aged ≥ 65 years, females, and stroke cases characterised by minor symptoms demonstrated a significantly increased stroke risk due to the effects of warm-season cold spells [66].

### Heart failure

Compared with IHD and stroke, research on temperature and heart failure (HF) is less extensive, and few studies distinguish between HF phenotypes (HFrEF vs. HFpEF). Nevertheless, seasonal increases in HF admissions and mortality during winter months are well documented [67, 68]. The MCC analysis revealed that extreme heat led to 2.6 excess deaths per 1,000 HF deaths and a 12% overall increase in HF-related mortality [4]. (Fig. 4)

A nationwide Swedish study indicated that short-term exposure to both low and high temperatures was associated with an increased risk of all-cause and cardiovascular mortality in patients with heart failure. In addition, the analysis stratified by time periods showed that the mortality risk associated with high temperatures appeared to be increasing over time [59].

An extensive Japanese study found that low temperatures significantly increased the risk of HF hospitalisation, with an RR of 1.571 (95% CI: 1.487–1.660). This effect was substantially greater than that observed for IHD (RR 1.119) or stroke (RR 1.107) [69]. Extreme heat also increased HF risk, though the magnitude was smaller (RR 1.030, 95% CI: 1.007–1.054). Older adults, particularly those aged ≥ 85 years, were especially vulnerable. Given the interplay between thermoregulation, neurohormonal activation, fluid balance, and comorbid renal disease, HF patients may experience severe physiological stress during both heat and cold events.

### Arrhythmias

The relationship between non-optimal temperatures and arrhythmias is complex and less consistent than for ischemic events or stroke. This inconsistency may stem from the use of broad arrhythmia categories rather than distinguishing between atrial and ventricular arrhythmias. In a South Korean study, each 1 °C increase in diurnal temperature range was associated with a 1.84% (95% CI: 0.34–3.37%) increase in [70] At the same time, a separate analysis showed that a 1 °C rise in the same-day temperature increased the odds of ventricular ectopy by 1.10 (95% CI: 1.04–1.17) [71].

However, larger datasets have reported null findings. A study of 345,052 arrhythmia admissions in Ontario found no association between temperature extremes and arrhythmia-related outcomes [72]. Likewise, an investigation in London observed no increase in defibrillator activations during periods of higher temperatures but for low temperatures [73]. To clarify these correlations, future studies must analyse specific arrhythmia phenotypes, incorporate continuous exposure data (especially night-time temperatures), and apply patient-level electrocardiographic monitoring.

## Interactions between air pollution, non-optimal temperatures, and cardiovascular health

Air pollution has been recognized as a human health risk since antiquity, but its composition, sources, and toxicological complexity have changed dramatically with industrialization and urbanization [74]. Climate, weather and air quality are deeply intertwined: climate and weather strongly influence pollutant formation, transport, and dispersion, while anthropogenic air pollution is itself a major driver of climate change [75]. The cardiovascular effects of air pollution are associated with its intricate chemical properties and particle size distributions, both of which depend on emission sources and prevailing meteorological conditions [42, 76]. Because weather and climate substantially shape atmospheric chemistry, long-standing assumptions about air-pollution health effects must be continually reassessed in the context of rapid global change. Although the detailed chemistry of air pollution has been comprehensively reviewed elsewhere, it is important to emphasize that both particulate matter and gaseous pollutants contribute substantially to cardiovascular toxicity [77–82]. Recent global estimates indicate that exposure to PM₂.₅ and ozone accounts for approximately 8.4 million premature deaths annually [83], and PM₂.₅ exposure alone reduces global life expectancy by two to three years [84]. Air pollution is considered a leading global health risk. It accounts for more than one in eight deaths worldwide, contributing to 7.9 million lives lost in 2023 alone. As a leading risk factor for early death, on par with high blood pressure, air pollution drives a massive burden of non-communicable diseases: 86% of pollution-related deaths are linked to conditions such as heart disease, stroke, COPD, diabetes, and dementia, particularly in adults over 60 [85].

Temperature profoundly modifies air-pollution dynamics. Higher temperatures enhance photochemical reactions, promoting the formation of ozone and secondary aerosols while reducing atmospheric mixing and pollutant dispersion [4]. Rising temperatures also increase chemical reaction rates in the atmosphere, facilitating the production of sulphate, organics and other secondary particles [86–88]. Conversely, temperature inversions trap pollutants close to the ground, creating episodes of extreme air pollution that intensify cardiovascular toxicity, often coinciding with heat extremes. The interaction between air pollution and temperature is increasingly recognized as a critical modifier of cardiovascular risk. The “climate penalty” describes the phenomenon in which rising temperatures accelerate the formation of secondary pollutants, such as ozone. Numerous studies demonstrate that both short-term and long-term ozone exposure increases cardiovascular mortality, with stronger associations observed during periods of elevated temperature [89–91]. A time-series study in eight European cities found that pollution-related cardiovascular and all-cause mortality was more pronounced on hot days [92]. Ozone-related mortality increased significantly under warmer conditions. Mechanistically, the synergy between heat and air pollution likely reflects overlapping biological pathways, including enhanced systemic oxidative stress, vascular inflammation, endothelial dysfunction, and thrombogenicity.

A central shared pathway through which both heat and air pollution exert cardiovascular toxicity is activation of the hypothalamic–pituitary–adrenal (HPA) axis. Heat stress engages the HPA axis through hypothalamic thermoreceptor signalling, increases in core body temperature, and concomitant sympathetic activation, leading to enhanced release of corticotropin-releasing hormone, adrenocorticotropic hormone, cortisol, and catecholamines [93] Air pollutants, particularly fine and ultrafine particulate matter and ozone, activate the HPA axis through complementary mechanisms, including pulmonary and systemic oxidative stress and inflammation, neuroinflammatory signalling via the olfactory and vagal pathways [94], and direct translocation of ultrafine particles into the central nervous system [95] all converging on hypothalamic stress centres. Sustained HPA activation in response to both stressors raises circulating cortisol and catecholamine levels, promoting tachycardia, hypertension, autonomic imbalance with reduced heart-rate variability, insulin resistance, dyslipidaemia, endothelial dysfunction, platelet activation, and a prothrombotic and pro-inflammatory milieu [96]. When heat and air pollution co-occur, this neuroendocrine stress response is amplified, providing a unifying mechanistic framework for the synergistic cardiovascular toxicity observed in epidemiological studies [5].

A systematic review reported strong interactive effects between heat and air pollutants, especially ozone and PM₂.₅, with consistent evidence for elevated all-cause and cardiovascular mortality under co-exposure [97]. Real-world studies support these findings. In California, the combined impact of extreme heat and PM₂.₅ exceeded the sum of their independent effects by far [98]. All-cause mortality increased by 6.1% on extreme heat–only days and 5.0% on extreme PM₂.₅-only days, but by 21.0% on days when both exposures co-occurred. Cardiovascular and respiratory mortality rose even more: 29.9% and 38.0%, respectively. An extensive case-crossover study of 202,678 MI deaths in Jiangsu Province, China, showed that both heatwaves and cold spells increased MI mortality (ORs ranging from 1.18 to 1.74 for heat and 1.04–1.12 for freezing). PM₂.₅ exposure on the preceding day further increased MI mortality risk; up to 2.8% of MI deaths were attributable to combined exposure to extreme temperatures and PM₂.₅ above WHO interim target 3 (37.5 µg/m³) [87]. In addition, co-exposure to high temperatures and PM₁₀, PM₂.₅, ozone, or NO₂ further increased cardiovascular risk, with the strongest interactions observed for ozone and NO₂ [86].

**Fig. 5:**
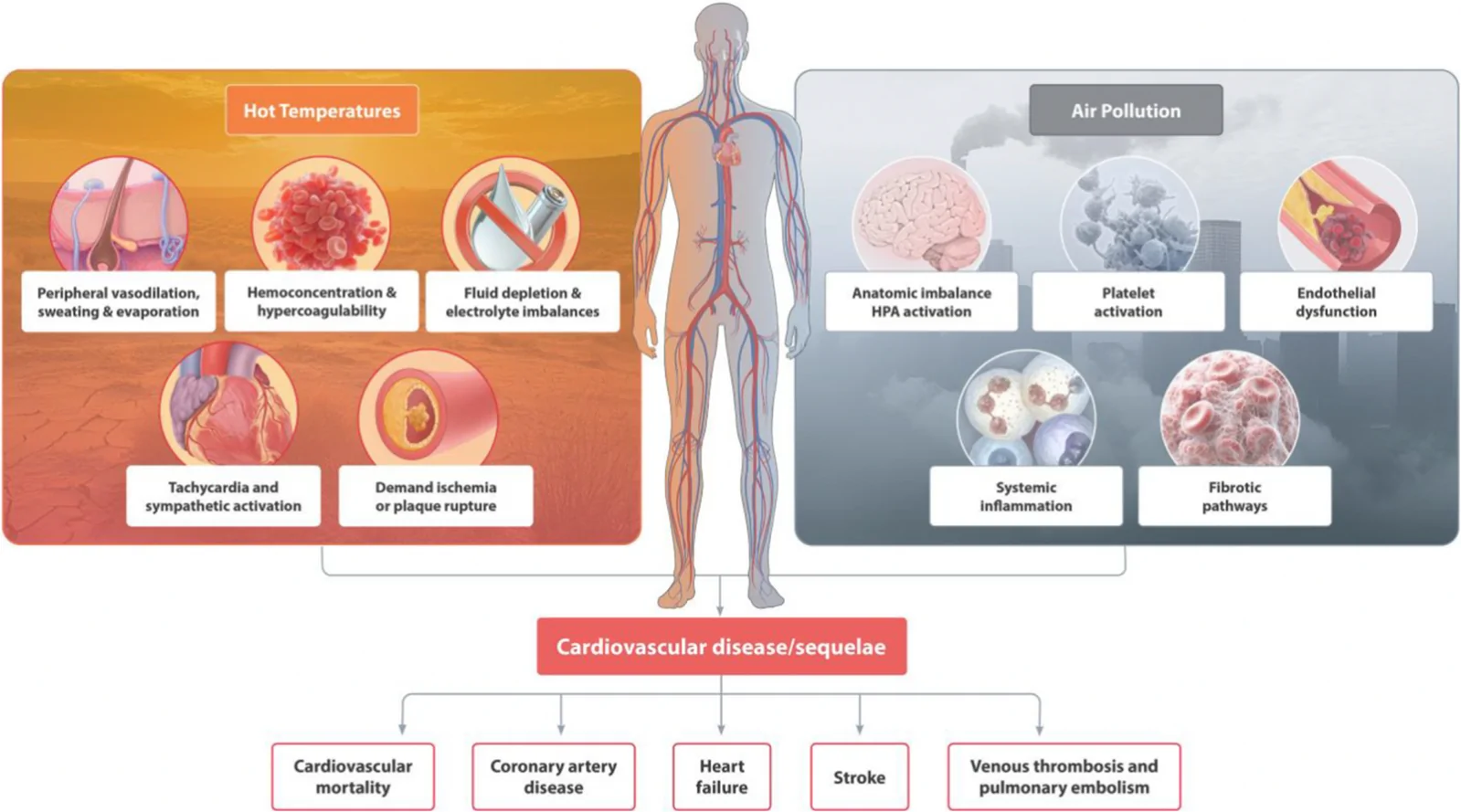
Synergistic cardiovascular effects of hot temperatures and air pollution. This figure illustrates the parallel and overlapping biological pathways through which extreme heat and air pollution jointly increase cardiovascular risk. High temperatures (left) trigger peripheral vasodilation with increased sweating and evaporative loss, haemoconcentration and hypercoagulability, fluid depletion and electrolyte imbalance, tachycardia with sympathetic activation, and demand ischaemia or plaque rupture. Air pollution (right) activates multiple toxicological pathways, including hypothalamic–pituitary–adrenal (HPA) axis imbalance, platelet activation, endothelial dysfunction, systemic inflammation, and pro-fibrotic signalling. Together, these stressors converge on shared cardiovascular mechanisms, autonomic imbalance, oxidative stress, vascular injury, thrombogenicity, and impaired myocardial oxygen supply–demand balance, culminating in a spectrum of adverse outcomes. These include cardiovascular mortality such as ischemic heart disease, heart failure, stroke, **and** venous thrombosis or pulmonary embolism. The figure underscores the compounding health burden created when rising global temperatures and persistent air pollution co-occur, particularly affecting vulnerable populations. (with permission from [100])

These findings underscore a central public health implication: even if the relative risks of heat or pollution appear modest at an individual level, the population-level burden is enormous due to widespread exposure. A recent European Union white paper emphasised the substantial co-benefits of integrating air-pollution mitigation into heat-health protection strategies, highlighting the opportunity to reduce cardiovascular morbidity and mortality significantly [99]. Figure 5 outlines schematically the distinct and overlapping biological pathways through which air pollution and non-optimal temperatures contribute to cardiovascular injury.

## Heat-related cardiovascular risk in vulnerable populations

**Fig. 6:**
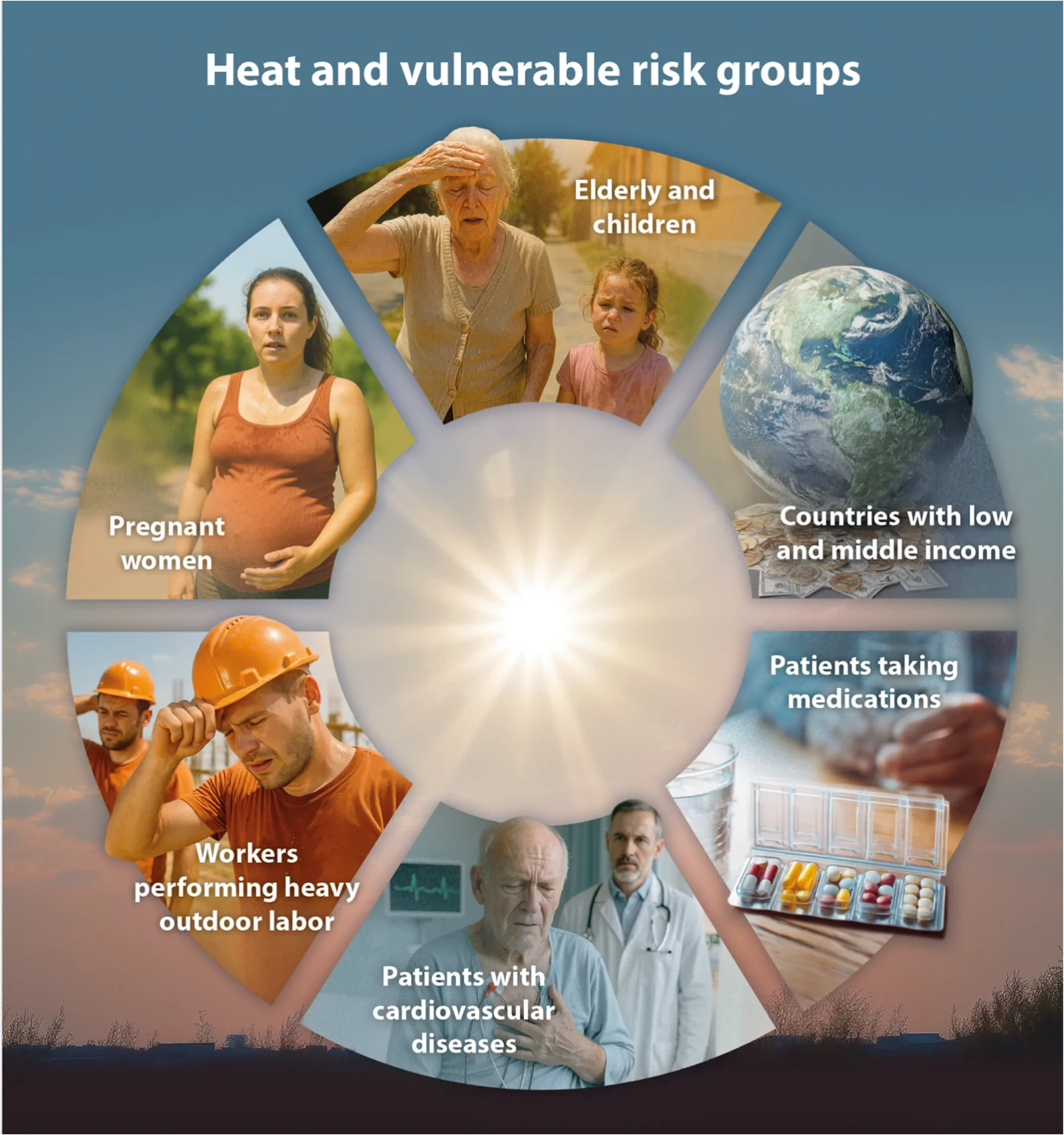
Vulnerable population groups at heightened risk during heat exposure. This schematic illustrates key groups that are particularly susceptible to the adverse health effects of elevated ambient temperatures. Elderly individuals and young children have impaired thermoregulation; pregnant women face increased metabolic and cardiovascular strain; and patients with cardiovascular disease are prone to heat-induced decompensation. People taking medications, such as diuretics, antihypertensives, anticholinergics, or psychotropics, may experience altered fluid balance or impaired heat dissipation. Workers performing heavy outdoor labour are exposed to extreme physical and environmental stress. Populations in low- and middle-income countries often lack infrastructure, cooling capacity, and access to healthcare, further amplifying heat-related risks. Collectively, these groups require targeted prevention strategies, early warning systems, and tailored adaptation measures to mitigate heat-related morbidity and mortality. The graphic generated using Midjourney and ChatGPT/DALL-E, and edited and laid out using Adobe Firefly, Adobe Photoshop, and Adobe InDesign

Social inequalities in exposure to extreme heat have been documented globally, and evidence from all global regions indicates that disadvantaged social groups have lower adaptive capacity, meaning that they are less able to avoid heat or protect themselves from it [101]. Social factors such as socio-economic status, neighbourhood environment, workplace and work characteristics, and race or ethnicity are also likely to be related to unequal exposures to further environmental risks such as air pollution, noise, and a lack of green space (Fig. 6).

A substantial body of epidemiological evidence shows that cardiovascular risks associated with heat exposure are disproportionately concentrated among older adults, individuals with pre-existing chronic conditions, and socioeconomically disadvantaged communities [102]. Heat vulnerability indices, developed in multiple countries, integrate factors such as age, chronic disease burden, medication use, poverty, and housing characteristics to identify geographic areas [103].

Aging markedly reduces physiological resilience to heat. Older adults experience diminished sweating capacity, impaired vasodilatory responses, reduced cardiac reserve, and blunted thirst perception. Under hot and humid conditions (e.g., 42 °C, 30–72% humidity), these limitations substantially heighten cardiovascular risk. Meta-analytic data indicate that a 1 °C rise in temperature increases the relative risk of cardiovascular mortality by 0.8%, especially in people aged ≥ 65 years (1.7% vs. 0.9% in younger adults) [9]. Country-specific data echo this pattern: in Germany, heat-related mortality was 5% higher in adults aged 65–74 and 17% higher in those ≥ 75 years compared with individuals under 65 [104].

Disparities are even more striking across global income levels. Low- and middle-income countries (LMICs) experience some of the world’s highest summer temperatures and most intense heatwaves, yet often lack adequate infrastructure, such as reliable electricity, air conditioning, green space, and robust healthcare systems, to mitigate heat exposure [105]. A recent analysis found a 9% higher risk of cardiovascular mortality per 1 °C rise in LMICs relative to upper-middle-income nations [105]. Data from the MCC network further show that heat-related stroke and heart-failure mortality are higher in countries with low GDP per capita. In contrast, heat-related IHD mortality was slightly more pronounced in high-income settings [4].

Education and social disadvantage also modulate vulnerability. In Europe, older adults with lower educational attainment experienced significantly higher heat-related cardiovascular mortality [106]. In the United States, many climate-vulnerable urban neighbourhoods correspond to historically redlined communities that have endured decades of structural disinvestment, environmental inequality, and under-resourced health systems [107].

Further high-risk groups include residents of low-lying island nations, especially in the Pacific and Indian Oceans, as well as indigenous communities across Africa, Australia/New Zealand, and the Americas, who often face heightened exposure, limited adaptive capacity, and cultural or geographical barriers to accessing cooling and healthcare resources [108].

## Drug–heat interactions and their cardiovascular implications

Heat exposure not only imposes direct physiological stress but also interacts with commonly used medications, altering pharmacokinetics, pharmacodynamics, and thermoregulatory capacity. These interactions may heighten susceptibility to syncope, electrolyte imbalance, arrhythmias, renal dysfunction, and ischemic events, particularly in older adults and patients with multimorbidity.

Heat-induced vasodilation can potentiate the hypotensive effects of antihypertensive medications, increasing the risk of dizziness, falls, and myocardial ischemia [50]. Several drug classes interfere with thermoregulation. Anticholinergics reduce sweating, predisposing individuals to dangerous hyperthermia. Diuretics, widely prescribed for hypertension and heart failure, exacerbate dehydration and electrolyte disturbances during heatwaves. Beta-blockers blunt the compensatory increase in heart rate necessary to dissipate heat, impairing cardiovascular adjustment to thermal stress.

Epidemiological evidence highlights the clinical significance of these interactions. During the 2003 European heatwave, a matched case-control study of 1,405 emergency admissions in France found that anticholinergic medications independently increased the risk of hyperthermia or heatstroke [109]. Another study of 345 heatstroke admissions during the same event identified diuretic use as a predictor of higher in-hospital mortality [110]. A recent study [111] found a significantly increased risk of nonfatal myocardial infarction on hot days compared with cooler control days in patients taking antiplatelet medications or beta-blockers, compared with patients not taking these drugs. Antiplatelet use was associated with a 63% increase in risk, and beta-blocker use with a 65% increase in risk. Patients who took both medications had a 75% cumulated risk. Non-users of these medications were not more likely to have a heart attack on hot days. It is also interesting to note that the effect of medication use was stronger in the younger age group (25–59 years) than in older patients (60–74 years). However, the latter were more likely to have underlying coronary heart disease. These findings suggest that certain medications may shift an individual’s heat tolerance threshold, thereby increasing the likelihood of severe cardiovascular complications during heatwaves.

Given these risks, clinicians must adopt a proactive approach during extreme heat events. Strategies include patient education on hydration, symptom recognition, avoidance of outdoor activity during peak heat, and temporary adjustments to medication regimens in high-risk individuals. Further research is needed to clarify the thermal effects of other widely used medications, including antidepressants and antipsychotics, which may similarly impair thermoregulation and heighten cardiovascular vulnerability under heat stress.

## Heat-health action planning and clinical preparedness

WHO/Europe recommends that countries, regions, and municipalities develop Heat–Health Action Plans (HHAPs) to systematically prevent, manage, and mitigate heat-related health risks. HHAPs provide a coordinated public-health framework that spans multiple sectors and governance levels. According to WHO guidance, effective HHAPs incorporate eight core elements: a clearly designated lead agency, accurate alert and early-warning systems, coordinated communication strategies, measures to reduce indoor heat exposure, targeted protection for vulnerable groups, preparedness of health and social-care services, long-term urban planning, and continuous monitoring and evaluation. These actions should be implemented at national and institutional levels and be tightly linked to meteorological early-warning systems, with specific measures activated before and during heatwaves.

Given that many heat-vulnerable patients take medications for cardiovascular conditions, clinicians play a central role in HHAPs. As mentioned, frequently prescribed drugs, such as anticholinergics, antihypertensives, antiarrhythmics, antianginals, diuretics, antidepressants, insulin, and some analgesics, may lose efficacy, cause dehydration, impair thermoregulation, or increase the risk of adverse effects during heat exposure. Physicians must identify at-risk patients, provide behavioural guidance, assess medication regimens, and educate patients about proper drug storage (typically below 25 °C). Cardiologists are encouraged to integrate heat-specific counselling into routine care, conduct pre-summer risk assessments, and advise on hydration and medication adjustment strategies.

Further research is urgently needed to refine HHAPs and improve personalised heat-risk management. Priorities include enhancing early-warning and surveillance systems, evaluating the effectiveness of protective measures, and examining real-world drug–heat interactions. Wearable devices offer promise for monitoring individualised physiological responses, such as ambulatory blood pressure, arrhythmias, or dysglycaemia. They may help identify vulnerable patients, especially those with comorbidities such as diabetes or chronic respiratory disease. Systematic studies examining temperature effects on medication safety and pharmacodynamics remain critically lacking and should be a primary focus of future research.

## Regional, national, and global strategies for adaptation and health-system resilience

### Adaptation planning and strengthening resilience

Health systems occupy a central position in climate adaptation because they intersect directly with public health risks, disease prevention, emergency preparedness, and the delivery of care to vulnerable populations [21, 112]. The WHO Health System Resilience Framework and the new Operational Framework for Climate-Resilient and Low-Carbon Health Systems outline key components necessary to build climate-ready health infrastructures [113, 114]. These components include sustainable financing; strong governance and leadership; a trained and adaptable health workforce; robust service-delivery models; effective health information systems; resilient medical products and technologies; community engagement; and strategies to reduce the sector’s carbon footprint.

Understanding and implementing these elements is essential for evaluating and strengthening a health system’s adaptive capacity. Climate-resilient health systems can absorb shocks, maintain essential services for high-risk populations, and support long-term well-being despite environmental stressors. A recent scoping review found that most resilience-building efforts to date have occurred in high-income countries and have focused on investments in climate-resilient infrastructure, digital technologies, improved workforce training, and enhanced access to care for vulnerable groups [115]. However, global progress remains uneven, and only a minority of countries worldwide have implemented comprehensive adaptation and resilience strategies.

### Health policy measures

Strengthening health-system resilience requires integrating climate considerations into all levels of health policy and planning. This includes incorporating climate-related health risks into urban design, spatial planning, and environmental regulation, as well as embedding climate education into school curricula and health-professional training programs [116]. Policy measures should also promote heat-risk adaptation, coastal protection, early-warning systems, and national climate-action databases. Ensuring universal access to healthcare services during extreme events is central to these efforts.

Successful adaptation depends on long-term, multisectoral coordination; adequate and sustained funding; regular monitoring and evaluation; and strong regulatory enforcement. Unfortunately, many countries, particularly those most exposed to climate-related health hazards, still lack detailed, actionable policy frameworks to protect their populations.

### Health promotion and risk communication

Public education and risk communication are indispensable components of climate adaptation. Communities and health professionals must be informed about the health impacts of climate change, the role of environmental pollution, and the co-benefits of mitigation. Climate-informed health education programs should be widely accessible, culturally appropriate, and supported by community partnerships.

Effective adaptation also requires timely and targeted emergency communication, especially for high-risk groups. This includes real-time health messaging during extreme heat events, floods, or wildfire episodes, as well as rapid, disease-specific emergency response protocols. Strengthening these communication pathways improves preparedness, promotes early action, and reduces climate-related morbidity and mortality.

### Research gaps in heat–cardiovascular disease evidence

Despite mounting evidence that extreme heat increases cardiovascular mortality, major knowledge gaps continue to limit precise risk estimation, mechanistic understanding, and effective policy design (Table 2). Singh et al. identify several structural weaknesses in existing studies that must be addressed to advance the field.

**Table 2:** Key Research Gaps and Needed Advances in Heat/Cold–CVD Science

Gap	Description	Needed Solutions
1. Temporal variability	Unclear how heat/cold–CVD relationships change across decades and demographics.	Long time-series analyses with standardized methods.
2. Cause-specific outcomes	Limited evidence for MI, HF, arrhythmias, and stroke subtypes.	More granular ICD-based analyses; harmonic multi-country cohorts.
3. Environmental interactions	Synergistic effects with humidity, PM₂.₅, NO₂, O₃, and social vulnerability poorly understood.	Explicit interaction-modelling and environmental-justice frameworks.
4. Exposure misclassification	Overreliance on outdoor temperature; indoor/personal exposure largely missing.	Indoor heat monitoring; improved satellite, sensor integration.
5. Lack of LMIC data	Regions with highest burden have least research.	Investment in surveillance, meteorological–health data linkage.
6. Mechanistic uncertainty	Limited biological data connecting heat/cold to CVD pathways.	Mechanistic cohort studies with biomarkers and wearables.
7. Policy evaluation gaps	Effectiveness of heat/cold-health action plans is unclear.	Impact assessments, equity-focused evaluations.
8. Adaptation processes	Behavioural and physiological adaptation insufficiently studied.	Multi-year cohort studies across climates and SES groups.
9. Longterm	Repeated exposure to extreme events.	Multi-year cohort studies across effects and the impact on life expectancy climates and SES groups.

### Temporal changes in temperature–CVD associations

A key gap is the poor understanding of *how* exposure–response relationships evolve over time. While some regions show declining vulnerability due to improved adaptation or healthcare access, others demonstrate rising heat susceptibility, particularly among older adults and individuals with cardiovascular comorbidities. Most datasets are too short or inconsistent to characterize long-term trends robustly, and methodological differences impede comparability. More long-running analyses are urgently needed.

### Cause-specific cardiovascular mortality

Most studies continue to use aggregate CVD mortality, obscuring condition-specific risks. Evidence for myocardial infarction, arrhythmias, heart failure, and specific stroke subtypes remains limited and heterogeneous. Stroke appears especially sensitive to heat, yet findings vary by region and study design. Without more granular outcome data, including ICD-verified causes, the field cannot design tailored heat-health interventions.

### Interactions with other environmental and social stressors

Heat rarely acts alone. Humidity, air pollution (PM₂.₅, PM₁₀, NO₂, O₃), wind, vegetation, built environment characteristics, and social inequities jointly modify cardiovascular risk. Yet interaction effects are understudied, often inconsistently modelled, or treated only as confounders rather than mediators or effect modifiers. The synergistic effects of heat plus high air pollution appear substantial, but evidence remains mixed. Integrated environmental-justice frameworks that consider occupation, housing, SES, and urban–rural gradients are almost absent.

### Inadequate exposure assessment

Most studies rely on outdoor air temperature, even though people spend 80–90% of their time indoors. This introduces significant exposure misclassification, since indoor heat exposure (affected by insulation, air conditioning, household materials, and ventilation) can diverge sharply from ambient temperatures. High-resolution, standardised temperature datasets, especially in low- and middle-income countries (LMICs), are limited or absent. These regions face the most significant heat burden, yet contribute the least data.

### Limited evidence from LMICs and climate-vulnerable regions

Despite these regions bearing most of the heat-attributable cardiovascular mortality (e.g., South Asia, Africa, Eastern Mediterranean), high-quality epidemiological studies are scarce. Differences in baseline CVD rates, infrastructure, comorbidities, and adaptive capacity make extrapolation from high-income countries unreliable. Strengthening surveillance systems, vital statistics, and exposure-monitoring networks in LMICs is essential.

### Biological mechanisms and individual-level data

Current evidence links heat exposure to dehydration, haemoconcentration, sympathetic activation, and increased cardiac workload. But direct mechanistic evidence remains weak. Few studies integrate biomarkers, longitudinal cohorts, medication use, or clinical parameters (e.g., FMD, troponins, BNP). No randomised trials are feasible, but detailed longitudinal cohorts with wearable monitoring could transform understanding.

### Evaluation of policy effectiveness

Heat-health action plans are widely recommended, but their real-world impact remains unclear. Few studies evaluate whether interventions reduce mortality in vulnerable groups. Without rigorous assessment, policies risk remaining symbolic rather than protective.

## Summary and conclusion

Climate change has reshaped the global thermal environment, increasing the frequency, duration, and intensity of temperature extremes, with major consequences for cardiovascular health. While cold-related mortality still dominates the overall burden, heat-related cardiovascular mortality is rising rapidly. For Europe alone [8], projections under different emission and population scenarios estimate a temperature-related burden of 0.7–2 million deaths across the century, with rising heat-related deaths consistently outweighing the modest decline in cold-related mortality.

Chronic cardiovascular disease is particularly vulnerable to thermal stress because thermoregulation depends on tightly coordinated adjustments in blood flow, vascular tone, hydration, autonomic balance, and renal perfusion. Heat and cold disrupt these mechanisms, driving sympathetic activation, endothelial dysfunction, oxidative stress, haemoconcentration, prothrombotic shifts, renal injury, and myocardial oxygen supply–demand mismatch — converging on ischemic heart disease, stroke, heart-failure decompensation, and arrhythmias along the U- or J-shaped exposure–response curves seen across multi-country analyses.

A unifying mechanism is immune and neuroendocrine activation: heat triggers heat-shock protein release, NLRP3 inflammasome activation, and gut-barrier disruption with endotoxin translocation, while cold elicits sympathetic-driven leukocytosis, elevated IL-6/TNF-α, and increased susceptibility to respiratory infections. Both responses are amplified by concurrent HPA-axis activation, also engaged by air pollution, providing a coherent framework linking environmental exposure to the chronic inflammation, endothelial dysfunction, and plaque destabilisation underlying atherosclerotic disease.

Temperature does not act alone: air pollution, wildfires, dust storms, urban heat islands, and drug–heat interactions amplify cardiovascular risk, while social determinants concentrate it in older adults, patients with chronic CVD, outdoor workers, and populations in low- and middle-income countries. Important gaps remain regarding long-term temperature–CVD trends, cause-specific outcomes, interactions with pollution and social vulnerability, mechanistic immune–vascular pathways, and rigorous evaluation of heat- and cold-health action plans.

Clinically and politically, the message is clear: non-optimal temperature is a modifiable environmental risk factor for chronic cardiovascular disease. Integrating heat- and cold-risk assessment into cardiovascular care, strengthening climate-resilient health systems, and advancing mitigation and adaptation policies will deliver substantial co-benefits for global cardiovascular health in a warming and increasingly unstable climate.

## Data Availability

No datasets were generated or analysed during the current study.

## References

[1] Brief C (2025) State of the climate: 2025 on track to be second or third warmest year on record

[2] Adeministration NCfEINOaA (2026) Global Land and Ocean Averge Temperature Anomaly

[3] Alahmad B, Khraishah H, Althalji K, Borchert W, Al-Mulla F, Koutrakis P (2023) Connections Between Air Pollution, Climate Change, and Cardiovascular Health. Can J Cardiol 39:1182–1190 10.1016/j.cjca.2023.03.025 PMC11097327 37030516

[4] Alahmad B, Khraishah H, Roye D, Vicedo-Cabrera AM, Guo Y, Papatheodorou SI, Achilleos S, Acquaotta F, Armstrong B, Bell ML, Pan SC, de Sousa Zanotti Stagliorio Coelho M, Colistro V, Dang TN, Van Dung D, De’ Donato FK, Entezari A, Guo YL, Hashizume M, Honda Y, Indermitte E, Iniguez C, Jaakkola JJK, Kim H, Lavigne E, Lee W, Li S, Madureira J, Mayvaneh F, Orru H, Overcenco A, Ragettli MS, Ryti NRI, Saldiva PHN, Scovronick N, Seposo X, Sera F, Silva SP, Stafoggia M, Tobias A, Garshick E, Bernstein AS, Zanobetti A, Schwartz J, Gasparrini A, Koutrakis P (2023) Associations Between Extreme Temperatures and Cardiovascular Cause-Specific Mortality: Results From 27 Countries. Circulation 147:35–46 10.1161/CIRCULATIONAHA.122.061832 PMC9794133 36503273

[5] WHO (2023) Climate Change. Geneva, Switzerland

[6] Hay SI, Ong KL, Santomauro DF et al (2025) Burden of 375 diseases and injuries, risk-attributable burden of 88 risk factors, and healthy life expectancy in 204 countries and territories, including 660 subnational locations, 1990–2023: a systematic analysis for the Global Burden of Disease Study 2023. Lancet 406:1873–1922 10.1016/S0140-6736(25)01637-X PMC12535840 41092926

[7] Zhao Q, Guo Y, Ye T, Gasparrini A, Tong S, Overcenco A, Urban A, Schneider A, Entezari A, Vicedo-Cabrera AM, Zanobetti A, Analitis A, Zeka A, Tobias A, Nunes B, Alahmad B, Armstrong B, Forsberg B, Pan SC, Iniguez C, Ameling C, De la Cruz Valencia C, Astrom C, Houthuijs D, Dung DV, Roye D, Indermitte E, Lavigne E, Mayvaneh F, Acquaotta F, de’ Donato F, Di Ruscio F, Sera F, Carrasco-Escobar G, Kan H, Orru H, Kim H, Holobaca IH, Kysely J, Madureira J, Schwartz J, Jaakkola JJK, Katsouyanni K, Hurtado Diaz M, Ragettli MS, Hashizume M, Pascal M, Ryti N, Scovronick N, Michelozzi N, Matus Correa P, Goodman P, Nascimento Saldiva P, Abrutzky PH, Osorio R, Rao S, Fratianni S, Dang S, Colistro TN, Huber V, Lee V, Seposo W, Honda X, Guo Y, Bell YL, Li ML S (2021) Global, regional, and national burden of mortality associated with non-optimal ambient temperatures from 2000 to 2019: a three-stage modelling study. Lancet Planet Health 5:e415–e25 de Sousa Zanotti Stagliorio Coelho M, Valdes Ortega 10.1016/S2542-5196(21)00081-4 34245712

[8] Masselot P, Mistry MN, Rao S, Huber V, Monteiro A, Samoli E, Stafoggia M, de’Donato F, Garcia-Leon D, Ciscar J-C, Feyen L, Schneider A, Katsouyanni K, Vicedo-Cabrera AM, Aunan K, Gasparrini A (2025) Estimating future heat-related and cold-related mortality under climate change, demographic and adaptation scenarios in 854 European cities. Nat Med 31:1294–1302 10.1038/s41591-024-03452-2 PMC12003192 39870815

[9] Liu J, Varghese BM, Hansen A, Zhang Y, Driscoll T, Morgan G, Dear K, Gourley M, Capon A, Bi P (2022) Heat exposure and cardiovascular health outcomes: a systematic review and meta-analysis. Lancet Planet Health 6:e484–e95 10.1016/S2542-5196(22)00117-6 35709806

[10] Chen K, de Schrijver E, Sivaraj S, Sera F, Scovronick N, Jiang L, Roye D, Lavigne E, Kyselý J, Urban A, Schneider A, Huber V, Madureira J, Mistry MN, Cvijanovic I, Gasparrini A, Vicedo-Cabrera AM (2024) Impact of population aging on future temperature-related mortality at different global warming levels. Nat Commun 15:1796 10.1038/s41467-024-45901-z PMC10899213 38413648

[11] Masselot P, Mistry MN, Rao S, Huber V, Monteiro A, Samoli E, Stafoggia M, de’Donato F, Garcia-Leon D, Ciscar JC, Feyen L, Schneider A, Katsouyanni K, Vicedo-Cabrera AM, Aunan K, Gasparrini A (2025) Estimating future heat-related and cold-related mortality under climate change, demographic and adaptation scenarios in 854 European cities. Nat Med 31:1294–1302 10.1038/s41591-024-03452-2 PMC12003192 39870815

[12] Rajagopalan S, Landrigan PJ (2021) Pollution and the Heart. N Engl J Med 385:1881–1892 10.1056/NEJMra2030281 34758254

[13] Bunker A, Wildenhain J, Vandenbergh A, Henschke N, Rocklov J, Hajat S, Sauerborn R (2016) Effects of air temperature on climate-sensitive mortality and morbidity outcomes in the elderly; a systematic review and meta-analysis of epidemiological evidence. EBioMedicine 6:258–68 10.1016/j.ebiom.2016.02.034 PMC4856745 27211569

[14] Khraishah H, Alahmad B, Ostergard RL Jr., AlAshqar A, Albaghdadi M, Vellanki N, Chowdhury MM, Al-Kindi SG, Zanobetti A, Gasparrini A, Rajagopalan S (2022) Climate change and cardiovascular disease: implications for global health. Nat Rev Cardiol 19:798–812 10.1038/s41569-022-00720-x 35672485

[15] Vaduganathan M, Mensah GA, Turco JV, Fuster V, Roth GA (2022) The Global Burden of Cardiovascular Diseases and Risk: A Compass for Future Health. J Am Coll Cardiol 80:2361–2371 10.1016/j.jacc.2022.11.005 36368511

[16] Hajat S (2017) Health effects of milder winters: a review of evidence from the United Kingdom. Environ Health 16:109 10.1186/s12940-017-0323-4 PMC5773863 29219101

[17] Yu X, Liu J, Yin P, Gao Y, He C, Kan H, Zhou M, Huo Y, Chen R (2025) Nonlinear relation between cardiac mortality and excess temperature in heatwaves: exposure response in 2.39 million patients. J Am Coll Cardiol. 10.1016/j.jacc.2025.01.034 40131259

[18] Wang J, Chen Y, Liao W, He G, Tett SFB, Yan Z, Zhai P, Feng J, Ma W, Huang C, Hu Y (2021) Anthropogenic emissions and urbanization increase risk of compound hot extremes in cities. Nat Clim Change 11:1084–1089

[19] Munzel T, Daiber A, Steven S, Tran LP, Ullmann E, Kossmann S, Schmidt FP, Oelze M, Xia N, Li H, Pinto A, Wild P, Pies K, Schmidt ER, Rapp S, Kroller-Schon S (2017) Effects of noise on vascular function, oxidative stress, and inflammation: mechanistic insight from studies in mice. Eur Heart J 38:2838–2849 10.1093/eurheartj/ehx081 PMC5837459 28329261

[20] Munzel T, Sorensen M, Hahad O, Nieuwenhuijsen M, Daiber A (2023) The contribution of the exposome to the burden of cardiovascular disease. Nat Rev Cardiol 20:651–669 10.1038/s41569-023-00873-3 37165157

[21] Romanello M, Di Napoli C, Drummond P (2024) The 2024 report of the Lancet Countdown on health and climate change: Protecting health in a world facing multiple crises. Lancet 404:1909–1964

[22] Vicedo-Cabrera AM, Scovronick N, Sera F, Royé D, Schneider R, Tobias A, Astrom C, Guo Y, Honda Y, Hondula DM, Abrutzky R, Tong S, de Sousa Zanotti Stagliorio Coelho M, Saldiva PHN, Lavigne E, Correa PM, Ortega NV, Kan H, Osorio S, Kyselý J, Urban A, Orru H, Indermitte E, Jaakkola JJK, Ryti N, Pascal M, Schneider A, Katsouyanni K, Samoli E, Mayvaneh F, Entezari A, Goodman P, Zeka A, Michelozzi P, de’Donato F, Hashizume M, Alahmad B, Diaz MH, De La Cruz Valencia C, Overcenco A, Houthuijs D, Ameling C, Rao S, Ruscio FD, Carrasco-Escobar G, Seposo X, Silva S, Madureira J, Holobaca IH, Fratianni S, Acquaotta F, Kim H, Lee W, Iniguez C, Forsberg B, Ragettli MS, Guo YLL, Chen BY, Li S, Armstrong B, Aleman A, Zanobetti A, Schwartz J, Dang TN, Dung DV, Gillett N, Haines A, Mengel M, Huber V, Gasparrini A (2021) The burden of heat-related mortality attributable to recent human-induced climate change. Nat Clim Chang 11:492–500 10.1038/s41558-021-01058-x PMC7611104 34221128

[23] Martínez-Solanas È, Quijal-Zamorano M, Achebak H, Petrova D, Robine JM, Herrmann FR, Rodó X, Ballester J (2021) Projections of temperature-attributable mortality in Europe: a time series analysis of 147 contiguous regions in 16 countries. Lancet Planet Health 5:e446–e54 10.1016/S2542-5196(21)00150-9 34245715

[24] Kazi DS, Katznelson E, Liu CL, Al-Roub NM, Chaudhary RS, Young DE, McNichol M, Mickley LJ, Kramer DB, Cascio WE, Bernstein AS, Rice MB (2024) Climate Change and Cardiovascular Health: A Systematic Review. JAMA Cardiol 9:748–757 10.1001/jamacardio.2024.1321 PMC11366109 38865135

[25] Yang J, Yin P, Zhou M, Ou CQ, Guo Y, Gasparrini A, Liu Y, Yue Y, Gu S, Sang S, Luan G, Sun Q, Liu Q (2015) Cardiovascular mortality risk attributable to ambient temperature in China. Heart 101:1966–1972 10.1136/heartjnl-2015-308062 26567233

[26] Silveira IH, Oliveira BFA, Cortes TR, Junger WL (2019) The effect of ambient temperature on cardiovascular mortality in 27 Brazilian cities. Sci Total Environ 691:996–1004 10.1016/j.scitotenv.2019.06.493 31326821

[27] Son JY, Lee JT, Anderson GB, Bell ML (2012) The impact of heat waves on mortality in seven major cities in Korea. Environ Health Perspect 120:566–571 10.1289/ehp.1103759 PMC3339449 22266672

[28] Dong W, Zeng Q, Ma Y, Li G, Pan X (2016) Impact of heat wave definitions on the added effect of heat waves on cardiovascular mortality in Beijing, China. Int J Environ Res Public Health. 10.3390/ijerph13090933 PMC5036765 27657103

[29] Liu J, Qi J, Yin P, Liu W, He C, Gao Y, Zhou L, Zhu Y, Kan H, Chen R, Zhou M (2024) Rising cause-specific mortality risk and burden of compound heatwaves amid climate change. Nat Clim Change 14:1201–1209

[30] Zhang X, Zhao L, Tong DQ, Wu G, Dan M, Teng B (2016) A Systematic Review of Global Desert Dust and Associated Human Health Effects. Atmosphere 7:158

[31] Hashizume M, Kim Y, Ng CFS, Chung Y, Madaniyazi L, Bell ML, Guo YL, Kan H, Honda Y, Yi SM, Kim H, Nishiwaki Y (2020) Health effects of Asian dust: a systematic review and meta-analysis. Environ Health Perspect 128:66001 10.1289/EHP5312 PMC7319773 32589456

[32] Chan CC, Chuang KJ, Chen WJ, Chang WT, Lee CT, Peng CM (2008) Increasing cardiopulmonary emergency visits by long-range transported Asian dust storms in Taiwan. Environ Res 106:393–400 10.1016/j.envres.2007.09.006 17959168

[33] Alahmad B, Khraishah H, Kamineni M, Royé D, Papatheodorou SI, Vicedo-Cabrera AM, Guo Y, Lavigne E, Armstrong B, Sera F, Bernstein AS, Zanobetti A, Garshick E, Schwartz J, Bell ML, Al-Mulla F, Koutrakis P, Gasparrini A (2024) Extreme Temperatures and Stroke Mortality: Evidence From a Multi-Country Analysis. Stroke 55:1847–1856 10.1161/STROKEAHA.123.045751 PMC11196199 38776169

[34] Imberti L, Tiecco G, Logiudice J, Castelli F, Quiros-Roldan E (2025) Effects of climate change on the immune system: a narrative review. Health Sci Rep 8:e70627 10.1002/hsr2.70627 PMC12007019 40256129

[35] Libby P (2021) The changing landscape of atherosclerosis. Nature 592:524–533 10.1038/s41586-021-03392-8 33883728

[36] Swirski FK, Nahrendorf M (2013) Leukocyte behavior in atherosclerosis, myocardial infarction, and heart failure. Science 339:161–166 10.1126/science.1230719 PMC3891792 23307733

[37] Bouchama A, Abuyassin B, Lehe C, Laitano O, Jay O, O’Connor FG, Leon LR (2022) Classic and exertional heatstroke. Nat Rev Dis Primers 8:8 10.1038/s41572-021-00334-6 35115565

[38] Leon LR, Bouchama A (2015) Heat stroke. Compr Physiol 5:611–647 10.1002/cphy.c140017 25880507

[39] Kregel KC (2002) Heat shock proteins: modifying factors in physiological stress responses and acquired thermotolerance. J Appl Physiol (1985) 92:2177–2186 10.1152/japplphysiol.01267.2001 11960972

[40] Janský L, Pospísilová D, Honzová S, Ulicný B, Srámek P, Zeman V, Kamínková J (1996) Immune system of cold-exposed and cold-adapted humans. Eur J Appl Physiol Occup Physiol 72:445–450 10.1007/BF00242274 8925815

[41] Li Y, Wu J, Xu Y, Dong J, Xing B, Wang Y, Zhou Z, Sun B, Li J, Yu L, Wang H (2025) Cold exposure and the cardiovascular system: from physiological adaptation to pathological risk. Front Physiol 16:1740919 10.3389/fphys.2025.1740919 PMC12819719 41574187

[42] Rajagopalan S, Al-Kindi SG, Brook RD (2018) Air Pollution and Cardiovascular Disease: JACC State-of-the-Art Review. J Am Coll Cardiol 72:2054–2070 10.1016/j.jacc.2018.07.099 30336830

[43] Kenny GP, Jay O (2013) Thermometry, calorimetry, and mean body temperature during heat stress. Compr Physiol 3:1689–1719 10.1002/cphy.c130011 24265242

[44] Fealey RD (2013) Interoception and autonomic nervous system reflexes thermoregulation. Handb Clin Neurol 117:79–88 10.1016/B978-0-444-53491-0.00007-9 24095117

[45] Ni W, Areal AT, Lechner K, Breitner S, Zhang S, Woeckel M, Slesinski SC, Nikolaou N, Dallavalle M, Schikowski T, Schneider A (2025) Low and high air temperature and cardiovascular risk. Atherosclerosis 406:119238 10.1016/j.atherosclerosis.2025.119238 40383648

[46] Rowell LB (1983) Cardiovascular aspects of human thermoregulation. Circ Res 52:367–379 10.1161/01.res.52.4.367 6339107

[47] Boyette LC, Manna B (2025) Physiology, myocardial oxygen demand. *StatPearls*. Treasure Island (FL): Editor: StatPearls Publishing Copyright © 2025. StatPearls Publishing LLC Pages: Pages: e1–eNBK499897 29763072

[48] Cheng J, Xu Z, Bambrick H, Prescott V, Wang N, Zhang Y, Su H, Tong S, Hu W (2019) Cardiorespiratory effects of heatwaves: a systematic review and meta-analysis of global epidemiological evidence. Environ Res 177:108610 10.1016/j.envres.2019.108610 31376629

[49] Roth GA, Johnson C, Abajobir A, Abd-Allah F, Abera SF et al (2017) Global, Regional, and National Burden of Cardiovascular Diseases for 10 Causes, 1990 to 2015. J Am Coll Cardiol 70:1–25 10.1016/j.jacc.2017.04.052 PMC5491406 28527533

[50] Ebi KL, Capon A, Berry P, Broderick C, de Dear R, Havenith G, Honda Y, Kovats RS, Ma W, Malik A, Morris NB, Nybo L, Seneviratne SI, Vanos J, Jay O (2021) Hot weather and heat extremes: health risks. Lancet 398:698–708 10.1016/S0140-6736(21)01208-3 34419205

[51] Filep EM, Murata Y, Endres BD, Kim G, Stearns RL, Casa DJ (2020) Exertional heat stroke, modality cooling rate, and survival outcomes: a systematic review. Medicina (Kaunas) 10.3390/medicina56110589 PMC7694459 33167534

[52] Tsuji B, Hayashi K, Kondo N, Nishiyasu T (2016) Characteristics of hyperthermia-induced hyperventilation in humans. Temp (Austin) 3:146–160 10.1080/23328940.2016.1143760 PMC4879782 27227102

[53] Dematte JE, O’Mara K, Buescher J, Whitney CG, Forsythe S, McNamee T, Adiga RB, Ndukwu IM (1998) Near-fatal heat stroke during the 1995 heat wave in Chicago. Ann Intern Med 129:173–181 10.7326/0003-4819-129-3-199808010-00001 9696724

[54] Khraishah H, Chen Z, Rajagopalan S (2024) Understanding the Cardiovascular and Metabolic Health Effects of Air Pollution in the Context of Cumulative Exposomic Impacts. Circ Res 134:1083–1097 10.1161/CIRCRESAHA.124.323673 PMC11253082 38662860

[55] Chen K, Breitner S, Wolf K, Hampel R, Meisinger C, Heier M, von Scheidt W, Kuch B, Peters A, Schneider A (2019) Temporal variations in the triggering of myocardial infarction by air temperature in Augsburg, Germany, 1987–2014. Eur Heart J 40:1600–1608 10.1093/eurheartj/ehz116 30859207

[56] Fuhrmann CM, Sugg MM, Konrad CE 2nd, Waller A (2016) Impact of extreme heat events on emergency department visits in North Carolina (2007–2011). J Community Health 41:146–56 10.1007/s10900-015-0080-7 26289379

[57] Basu R (2009) High ambient temperature and mortality: a review of epidemiologic studies from 2001 to 2008. Environ Health 8:40 10.1186/1476-069X-8-40 PMC2759912 19758453

[58] Yu W, Mengersen K, Wang X, Ye X, Guo Y, Pan X, Tong S (2012) Daily average temperature and mortality among the elderly: a meta-analysis and systematic review of epidemiological evidence. Int J Biometeorol 56:569–581 10.1007/s00484-011-0497-3 21975970

[59] Ni W, Stafoggia M, Zhang S, Ljungman P, Breitner S, de Bont J, Jernberg T, Atar D, Schneider A, Agewall S (2025) Short-term exposure to ambient temperature variability and myocardial infarction hospital admissions: a nationwide case-crossover study in Sweden. PLoS Med 22:e1004607 10.1371/journal.pmed.1004607 PMC12091774 40392899

[60] Ni W, Stafoggia M, Zhang S, Ljungman P, Breitner S, Bont J, Jernberg T, Atar D, Agewall S, Schneider A (2024) Short-Term Effects of Lower Air Temperature and Cold Spells on Myocardial Infarction Hospitalizations in Sweden. J Am Coll Cardiol 84:1149–1159 10.1016/j.jacc.2024.07.006 39230547

[61] Lian H, Ruan Y, Liang R, Liu X, Fan Z (2015) Short-Term Effect of Ambient Temperature and the Risk of Stroke: A Systematic Review and Meta-Analysis. Int J Environ Res Public Health 12:9068–9088 10.3390/ijerph120809068 PMC4555265 26264018

[62] Wang X, Cao Y, Hong D, Zheng D, Richtering S, Sandset EC, Leong TH, Arima H, Islam S, Salam A, Anderson C, Robinson T, Hackett ML (2016) Ambient temperature and stroke occurrence: a systematic review and meta-analysis. Int J Environ Res Public Health. 10.3390/ijerph13070698 PMC4962239 27420077

[63] Han MH, Yi HJ, Kim YS, Kim YS (2015) Effect of seasonal and monthly variation in weather and air pollution factors on stroke incidence in Seoul, Korea. Stroke 46:927–935 10.1161/STROKEAHA.114.007950 25669311

[64] He C, Breitner S, Zhang S, Huber V, Naumann M, Traidl-Hoffmann C, Hammel G, Peters A, Ertl M, Schneider A (2024) Nocturnal heat exposure and stroke risk. Eur Heart J 45:2158–2166 10.1093/eurheartj/ehae277 PMC11212822 38768958

[65] Zhou L, Chen K, Chen X, Jing Y, Ma Z, Bi J, Kinney PL (2017) Heat and mortality for ischemic and hemorrhagic stroke in 12 cities of Jiangsu Province, China. Sci Total Environ 601–602:271–277 10.1016/j.scitotenv.2017.05.169 28558275

[66] He C, Breitner S, Zhang S, Naumann M, Traidl-Hoffmann C, Hammel G, Peters A, Ertl M, Schneider A (2025) Stroke risk associated with cold spells occurring during the warm season. Environ Int 199:109514 10.1016/j.envint.2025.109514 40328088

[67] Inglis SC, Clark RA, Shakib S, Wong DT, Molaee P, Wilkinson D, Stewart S (2008) Hot summers and heart failure: seasonal variations in morbidity and mortality in Australian heart failure patients (1994–2005). Eur J Heart Fail 10:540–549 10.1016/j.ejheart.2008.03.008 18495533

[68] Stewart S, McIntyre K, Capewell S, McMurray JJ (2002) Heart failure in a cold climate. Seasonal variation in heart failure-related morbidity and mortality. J Am Coll Cardiol 39:760–766 10.1016/s0735-1097(02)01685-6 11869838

[69] Pan R, Okada A, Yamana H, Yasunaga H, Kumazawa R, Matsui H, Fushimi K, Honda Y, Kim Y (2023) Association between ambient temperature and cause-specific cardiovascular disease admissions in Japan: a nationwide study. Environ Res 225:115610 10.1016/j.envres.2023.115610 36871945

[70] Kim J, Kim H (2017) Influence of ambient temperature and diurnal temperature range on incidence of cardiac arrhythmias. Int J Biometeorol 61:407–416 10.1007/s00484-016-1221-0 27568189

[71] Zanobetti A, Coull BA, Kloog I, Sparrow D, Vokonas PS, Gold DR, Schwartz J (2017) Fine-scale spatial and temporal variation in temperature and arrhythmia episodes in the VA normative aging study. J Air Waste Manag Assoc 67:96–104 10.1080/10962247.2016.1252808 PMC5543304 28001123

[72] Bai L, Li Q, Wang J, Lavigne E, Gasparrini A, Copes R, Yagouti A, Burnett RT, Goldberg MS, Villeneuve PJ, Cakmak S, Chen H (2016) Hospitalizations from hypertensive diseases, diabetes, and arrhythmia in relation to low and high temperatures: population-based study. Sci Rep 6:30283 10.1038/srep30283 PMC4960559 27456033

[73] McGuinn L, Hajat S, Wilkinson P, Armstrong B, Anderson HR, Monk V, Harrison R (2013) Ambient temperature and activation of implantable cardioverter defibrillators. Int J Biometeorol 57:655–662 10.1007/s00484-012-0591-1 22990411

[74] Rajagopalan S, Narula J (2022) The Perils of Anthropogenic Air Pollution: When All Roads Led to Rome. J Am Coll Cardiol 80:1829–1832 10.1016/j.jacc.2022.09.015 36328693

[75] Kaufman JD, Elkind MSV, Bhatnagar A, Koehler K, Balmes JR, Sidney S, Burroughs Peña MS, Dockery DW, Hou L, Brook RD, Laden F, Rajagopalan S, Bishop Kendrick K, Turner JR (2020) Guidance to reduce the cardiovascular burden of ambient air pollutants: a policy statement from the American Heart Association. Circulation 142:e432–e47 10.1161/CIR.0000000000000930 33147996

[76] Brook RD, Rajagopalan S, Pope CA 3rd, Brook JR, Bhatnagar A, Diez-Roux AV, Holguin F, Hong Y, Luepker RV, Mittleman MA, Peters A, Siscovick D, Smith SC Jr., Whitsel L, Kaufman JD (2010) Particulate matter air pollution and cardiovascular disease: an update to the scientific statement from the American Heart Association. Circulation 121:2331–78 10.1161/CIR.0b013e3181dbece1 20458016

[77] Xu X, Yavar Z, Verdin M, Ying Z, Mihai G, Kampfrath T, Wang A, Zhong M, Lippmann M, Chen LC, Rajagopalan S, Sun Q (2010) Effect of early particulate air pollution exposure on obesity in mice: role of p47phox. Arterioscler Thromb Vasc Biol 30:2518–2527 10.1161/ATVBAHA.110.215350 PMC3065931 20864666

[78] Munzel T, Gori T, Al-Kindi S, Deanfield J, Lelieveld J, Daiber A, Rajagopalan S (2018) Effects of gaseous and solid constituents of air pollution on endothelial function. Eur Heart J 39:3543–50 10.1093/eurheartj/ehy481 PMC6174028 30124840

[79] Al-Kindi SG, Brook RD, Biswal S, Rajagopalan S (2020) Environmental determinants of cardiovascular disease: lessons learned from air pollution. Nat Rev Cardiol 17:656–672 10.1038/s41569-020-0371-2 PMC7492399 32382149

[80] Hahad O, Rajagopalan S, Lelieveld J, Sorensen M, Frenis K, Daiber A, Basner M, Nieuwenhuijsen M, Brook RD, Munzel T (2023) Noise and air pollution as risk factors for hypertension: part I-epidemiology. *Hypertension* 10.1161/HYPERTENSIONAHA.122.18732 PMC10330192 37073726

[81] Hahad O, Rajagopalan S, Lelieveld J, Sorensen M, Kuntic M, Daiber A, Basner M, Nieuwenhuijsen M, Brook RD, Munzel T (2023) Noise and air pollution as risk factors for hypertension: part II-pathophysiologic insight. *Hypertension* 10.1161/HYPERTENSIONAHA.123.20617 PMC10330112 37073733

[82] Rajagopalan S, Brook RD, Salerno P, Bourges-Sevenier B, Landrigan P, Nieuwenhuijsen MJ, Munzel T, Deo SV, Al-Kindi S (2024) Air pollution exposure and cardiometabolic risk. Lancet Diabetes Endocrinol 12:196–208 10.1016/S2213-8587(23)00361-3 PMC11264310 38310921

[83] Lelieveld J, Haines A, Burnett R, Tonne C, Klingmuller K, Munzel T, Pozzer A (2023) Air pollution deaths attributable to fossil fuels: observational and modelling study. BMJ 383:e077784 10.1136/bmj-2023-077784 PMC10686100 38030155

[84] Lelieveld J, Pozzer A, Poschl U, Fnais M, Haines A, Munzel T (2020) Loss of life expectancy from air pollution compared to other risk factors: a worldwide perspective. Cardiovasc Res 116:1910–7 10.1093/cvr/cvaa025 PMC7449554 32123898

[85] HEI (2024) State of global air report 2024

[86] Rai M, Stafoggia M, de’Donato F, Scortichini M, Zafeiratou S, Vazquez Fernandez L, Zhang S, Katsouyanni K, Samoli E, Rao S, Lavigne E, Guo Y, Kan H, Osorio S, Kysely J, Urban A, Orru H, Maasikmets M, Jaakkola JJK, Ryti N, Pascal M, Hashizume M, Fook Sheng Ng C, Alahmad B, Hurtado Diaz M, De la Cruz Valencia C, Nunes B, Madureira J, Scovronick N, Garland RM, Kim H, Lee W, Tobias A, Iniguez C, Forsberg B, Astrom C, Maria Vicedo-Cabrera A, Ragettli MS, Leon Guo YL, Pan SC, Li S, Gasparrini A, Sera F, Masselot P, Schwartz J, Zanobetti A, Bell ML, Schneider A, Breitner S (2023) Heat-related cardiorespiratory mortality: effect modification by air pollution across 482 cities from 24 countries. Environ Int 174:107825 10.1016/j.envint.2023.107825 36934570

[87] Xu R, Huang S, Shi C, Wang R, Liu T, Li Y, Zheng Y, Lv Z, Wei J, Sun H, Liu Y (2023) Extreme Temperature Events, Fine Particulate Matter, and Myocardial Infarction Mortality. Circulation 148:312–323 10.1161/CIRCULATIONAHA.122.063504 37486993

[88] Braga AL, Zanobetti A, Schwartz J (2002) The effect of weather on respiratory and cardiovascular deaths in 12 U.S. cities. Environ Health Perspect 110:859–863 10.1289/ehp.02110859 PMC1240983 12204818

[89] Turner MC, Jerrett M, Pope CA 3rd, Krewski D, Gapstur SM, Diver WR, Beckerman BS, Marshall JD, Su J, Crouse DL, Burnett RT (2016) Long-term ozone exposure and mortality in a large prospective study. Am J Respir Crit Care Med 193:1134–42 10.1164/rccm.201508-1633OC PMC4872664 26680605

[90] Lim CC, Hayes RB, Ahn J, Shao Y, Silverman DT, Jones RR, Garcia C, Bell ML, Thurston GD (2019) Long-Term Exposure to Ozone and Cause-Specific Mortality Risk in the United States. Am J Respir Crit Care Med 200:1022–1031 10.1164/rccm.201806-1161OC PMC6794108 31051079

[91] Niu Y, Zhou Y, Chen R, Yin P, Meng X, Wang W, Liu C, Ji JS, Qiu Y, Kan H, Zhou M (2022) Long-term exposure to ozone and cardiovascular mortality in China: a nationwide cohort study. Lancet Planet Health 6:e496–e503 10.1016/S2542-5196(22)00093-6 35709807

[92] Chen K, Wolf K, Breitner S, Gasparrini A, Stafoggia M, Samoli E, Andersen ZJ, Bero-Bedada G, Bellander T, Hennig F, Jacquemin B, Pekkanen J, Hampel R, Cyrys J, Peters A, Schneider A (2018) Two-way effect modifications of air pollution and air temperature on total natural and cardiovascular mortality in eight European urban areas. Environ Int 116:186–196 10.1016/j.envint.2018.04.021 29689465

[93] Hannan FM, Leow MKS, Lee JKW, Kovats S, Elajnaf T, Kennedy SH, Thakker RV (2024) Endocrine effects of heat exposure and relevance to climate change. Nat Rev Endocrinol 20:673–684 10.1038/s41574-024-01017-4 39080505

[94] Thomson EM (2019) Air Pollution, Stress, and Allostatic Load: Linking Systemic and Central Nervous System Impacts. J Alzheimers Dis 69:597–614 10.3233/JAD-190015 PMC6598002 31127781

[95] Oberdörster G, Sharp Z, Atudorei V, Elder A, Gelein R, Kreyling W, Cox C (2004) Translocation of inhaled ultrafine particles to the brain. Inhal Toxicol 16:437–45 10.1080/08958370490439597 15204759

[96] Burford NG, Webster NA, Cruz-Topete D (2017) Hypothalamic-pituitary-adrenal axis modulation of glucocorticoids in the cardiovascular system. Int J Mol Sci. 10.3390/ijms18102150 PMC5666832 29035323

[97] Anenberg SC, Haines S, Wang E, Nassikas N, Kinney PL (2020) Synergistic health effects of air pollution, temperature, and pollen exposure: a systematic review of epidemiological evidence. Environ Health 19:130 10.1186/s12940-020-00681-z PMC7720572 33287833

[98] Rahman MM, McConnell R, Schlaerth H, Ko J, Silva S, Lurmann FW, Palinkas L, Johnston J, Hurlburt M, Yin H, Ban-Weiss G, Garcia E (2022) The Effects of Coexposure to Extremes of Heat and Particulate Air Pollution on Mortality in California: Implications for Climate Change. Am J Respir Crit Care Med 206:1117–1127 10.1164/rccm.202204-0657OC PMC9704834 35727303

[99] EU (2024) EXHAUSTION White Paper - Exposure to heat and air pollution in Europe. Important findings and recommendations

[100] Munzel T, Khraishah H, Schneider A, Lelieveld J, Daiber A, Rajagopalan S (2024) Challenges posed by climate hazards to cardiovascular health and cardiac intensive care: implications for mitigation and adaptation. Eur Heart J Acute Cardiovasc Care 13:731–44 10.1093/ehjacc/zuae113 PMC11518858 39468673

[101] Slesinski SC, Matthies-Wiesler F, Breitner-Busch S, Gussmann G, Schneider A (2025) Social inequalities in exposure to heat stress and related adaptive capacity: a systematic review. Environ Res Lett 20:033005. 10.1088/1748-9326/adb509

[102] Singh N, Areal AT, Breitner S, Zhang S, Agewall S, Schikowski T, Schneider A (2024) Heat and Cardiovascular Mortality: An Epidemiological Perspective. Circ Res 134:1098–1112 10.1161/CIRCRESAHA.123.323615 PMC11042530 38662866

[103] Bao J, Li X, Yu C (2015) The Construction and Validation of the Heat Vulnerability Index, a Review. Int J Environ Res Public Health 12:7220–7234 10.3390/ijerph120707220 PMC4515652 26132476

[104] Rai M, Breitner S, Huber V, Zhang S, Peters A, Schneider A (2023) Temporal variation in the association between temperature and cause-specific mortality in 15 German cities. Environ Res 229:115668 10.1016/j.envres.2023.115668 36958378

[105] Campbell S, Remenyi TA, White CJ, Johnston FH (2018) Heatwave and health impact research: a global review. Health Place 53:210–8 10.1016/j.healthplace.2018.08.017 30189362

[106] Saucy A, Ragettli MS, Vienneau D, de Hoogh K, Tangermann L, Schaffer B, Wunderli JM, Probst-Hensch N, Roosli M (2021) The role of extreme temperature in cause-specific acute cardiovascular mortality in Switzerland: a case-crossover study. Sci Total Environ 790:147958 10.1016/j.scitotenv.2021.147958 34098271

[107] Khadke S, Kumar A, Al-Kindi S, Rajagopalan S, Kong Y, Nasir K, Ahmad J, Adamkiewicz G, Delaney S, Nohria A, Dani SS, Ganatra S (2024) Association of environmental injustice and cardiovascular diseases and risk factors in the United States. J Am Heart Assoc 13:e033428 10.1161/JAHA.123.033428 PMC11179791 38533798

[108] Redvers N, Aubrey P, Celidwen Y, Hill K (2023) Indigenous peoples: traditional knowledges, climate change, and health. PLoS Glob Public Health 3:e0002474 10.1371/journal.pgph.0002474 PMC10575522 37831713

[109] Martin-Latry K, Goumy MP, Latry P, Gabinski C, Begaud B, Faure I, Verdoux H (2007) Psychotropic drugs use and risk of heat-related hospitalisation. Eur Psychiatry 22:335–338 10.1016/j.eurpsy.2007.03.007 17513091

[110] Misset B, De Jonghe B, Bastuji-Garin S, Gattolliat O, Boughrara E, Annane D, Hausfater P, Garrouste-Orgeas M, Carlet J (2006) Mortality of patients with heatstroke admitted to intensive care units during the 2003 heat wave in France: a national multiple-center risk-factor study. Crit Care Med 34:1087–92 10.1097/01.CCM.0000206469.33615.02 16484920

[111] Chen K, Dubrow R, Breitner S, Wolf K, Linseisen J, Schmitz T, Heier M, Scheidt W, Kuch B, Meisinger C, Peters A, Schwettmann L, Leidl R, Linkohr B, Grallert H, Gieger C, Schneider A (2022) Triggering of myocardial infarction by heat exposure is modified by medication intake. Nat Cardiovasc Res 1:1–5 10.1038/s44161-022-00102-z 39196082

[112] van Daalen KR, Romanello M, Rocklöv J, Semenza JC, Tonne C, Markandya A, Dasandi N, Jankin S, Achebak H, Ballester J, Bechara H, Callaghan MW, Chambers J, Dasgupta S, Drummond P, Farooq Z, Gasparyan O, Gonzalez-Reviriego N, Hamilton I, Hänninen R, Kazmierczak A, Kendrovski V, Kennard H, Kiesewetter G, Lloyd SJ, Lotto Batista M, Martinez-Urtaza J, Milà C, Minx JC, Nieuwenhuijsen M, Palamarchuk J, Quijal-Zamorano M, Robinson EJZ, Scamman D, Schmoll O, Sewe MO, Sjödin H, Sofiev M, Solaraju-Murali B, Springmann M, Triñanes J, Anto JM, Nilsson M, Lowe R (2022) The 2022 Europe report of the Lancet Countdown on health and climate change: towards a climate resilient future. Lancet Public Health 7:e942–e65 10.1016/S2468-2667(22)00197-9 PMC9597587 36306805

[113] WHO (2015) Operational framework for building climate resilient health systems

[114] WHO (2023) Operational framework for building climate resilient and low carbon health systems. Geneva, Switzerland

[115] Ansah EW, Amoadu M, Obeng P, Sarfo JO (2024) Health systems response to climate change adaptation: a scoping review of global evidence. BMC Public Health 24:2015 10.1186/s12889-024-19459-w PMC11285469 39075368

[116] Rajagopalan S, Ramaswami A, Bhatnagar A, Brook RD, Fenton M, Gardner C, Neff R, Russell AG, Seto KC, Whitsel LP, American Heart Association Council on H, Council on L, Cardiometabolic H, Council on Peripheral Vascular D, Council on Lifelong Congenital Heart D, American Heart Association Advocacy Coordinating C (2024) Toward Heart-Healthy and Sustainable Cities: A Policy Statement From the American Heart Association. Circulation 149:e1067–e89 Heart Health in the Y, Council on Cardiovascular S, Anesthesia, the 10.1161/CIR.0000000000001217 PMC12160618 38436070

